# HPV16 variants distribution in invasive cancers of the cervix, vulva, vagina, penis, and anus

**DOI:** 10.1002/cam4.870

**Published:** 2016-09-21

**Authors:** Sara Nicolás‐Párraga, Carolina Gandini, Ville N. Pimenoff, Laia Alemany, Silvia de Sanjosé, F. Xavier Bosch, Ignacio G. Bravo, the RIS HPV TT and HPV VVAP study groups

**Affiliations:** ^1^ Infections and Cancer UnitCancer Epidemiology Research ProgramCatalan Institute of Oncology (ICO)BarcelonaSpain; ^2^ Bellvitge Institute of Biomedical Research (IDIBELL)BarcelonaSpain; ^3^ CIBER in Epidemiology and Public Health (CIBERESP)BarcelonaSpain; ^4^ MIVEGECNational Center for Scientific Research (CNRS)MontpellierFrance

**Keywords:** Anogenital cancers, papillomavirus infection and cancer, viral diversity, viral evolution, virus–host interactions

## Abstract

Human papillomavirus (HPV)16 is the most oncogenic human papillomavirus, responsible for most papillomavirus‐induced anogenital cancers. We have explored by sequencing and phylogenetic analysis the viral variant lineages present in 692 HPV16‐monoinfected invasive anogenital cancers from Europe, Asia, and Central/South America. We have assessed the contribution of geography and anatomy to the differential prevalence of HPV16 variants and to the nonsynonymous *E6* T350G polymorphism. Most (68%) of the variance in the distribution of HPV16 variants was accounted for by the differential abundance of the different viral lineages. The most prevalent variant (above 70% prevalence) in all regions and in all locations was HPV16_A1‐3, except in Asia, where HPV16_A4 predominated in anal cancers. The differential prevalence of variants as a function of geographical origin explained 9% of the variance, and the differential prevalence of variants as a function of anatomical location accounted for less than 3% of the variance. Despite containing similar repertoires of HPV16 variants, we confirm the worldwide trend of cervical cancers being diagnosed significantly earlier than other anogenital cancers (early fifties vs. early sixties). Frequencies for alleles in the HPV16 *E6* T350G polymorphism were similar across anogenital cancers from the same geographical origin. Interestingly, anogenital cancers from Central/South America displayed higher 350G allele frequencies also within HPV16_A1‐3 lineage compared with Europe. Our results demonstrate ample variation in HPV16 variants prevalence in anogenital cancers, which is partly explained by the geographical origin of the sample and only marginally explained by the anatomical location of the lesion, suggesting that tissue specialization is not essential evolutionary forces shaping HPV16 diversity in anogenital cancers.

## Introduction

Certain human papillomaviruses (HPVs) are associated with certain human tumors. Based on the differential risk and association between infections and invasive cervical cancer (ICC), HPVs are classified into different risk groups 1. A number of HPVs are considered carcinogenic for humans (group 1) or possibly/probably carcinogenic (groups 2a and 2b, respectively) and are commonly referred to as “high‐risk HPVs” 1, 2. ICC is worldwide the second most common cancer affecting women and responsible for approximately 266,000 deaths per year (http://globocan.iarc.fr/Default.aspx). Persistent infection by oncogenic HPVs is considered a pre‐requisite for the development of virtually all ICCs 3. For this reason, the most extensive studies on HPVs have addressed cervical lesions and tumors 4. A similar repertoire of HPVs may also be responsible for different fractions of other anogenital tumors 5, as viral DNA has been detected in malignant proliferations in the penis (33% prevalence) 6, 7, anus (88% prevalence) 7, 8, vulva (29% prevalence) 9, and vagina (74% prevalence) 10.

Oncogenic potential is not evenly distributed among oncogenic HPVs. Instead, HPV prevalence largely differs between types and between geographical regions, and the probability of progression from a clinically asymptomatic cervical infection to ICC is different for different HPVs 11, 12. HPV16 is the most frequently detected HPV in all cervical infections, from normal cytology to ICC, in all world regions 11, 12. HPV16 is also the most oncogenic HPV, responsible for 61% of all ICCs worldwide 4 and for even higher fractions of other HPV‐associated anogenital carcinomas 6, 9, 10. The biological reasons underlying the increased prevalence and oncogenicity of HPV16 compared with other closely related viruses, for example, the sister viruses HPV31 and HPV35, remain unclear 13.

Sequence diversity within HPV types is described in terms of viral variants 14. The best classification for HPV16 variants has been proposed by Burk and coworkers, describing four lineages and a number of sublineages and applying an alphanumeric code, for example, HPV16_A4 15. Further, a large body of experimental research on the differential biological activities of HPV16 variants has focused on the *E6* gene, especially on the T350G polymorphism, corresponding to the L83V amino acid substitution in the E6 oncoprotein. The initial literature described 350T as the “prototype,” found in the “European” HPV16 variant, and the 350G allele as the “nonprototype,” found in “non‐European” HPV16 variants. However, the T350G polymorphism is found in different HPV16 variant lineages and is not a specific marker of any of them 16.

Papillomavirus variants are genetically very close, with above 98% nucleotide identity 14, but nonetheless HPV16 variants are suggested to differ in their oncogenic potential 17. Particularly, the *E6* T350G polymorphism has been associated with differential persistence and risk of progression to precancerous cervical lesions 17, 18.

The objective of this study was to characterize the viral component in a comprehensive set of invasive tumors of the cervix, vulva, vagina, penis, and anus, encompassing 35 countries within three continents. We aimed to analyze the differential prevalence of HPV16 variants as well as of the intensively studied T350G polymorphism as a function of the anatomical location of the lesion, the geographical origin of the samples, and the age at cancer diagnosis.

## Materials and Methods

### Samples

Samples analyzed in this study stem from a formalin‐fixed paraffin‐embedded (FFPE) sample repository from the Catalan Institute of Oncology (ICO), Barcelona, Spain, designed and constructed for the assessment of HPVs contribution to a number of anogenital human tumors 4, 6, 8, 9, 10. All samples were tested for the presence of tumor tissue and for the presence of HPVs DNA using the SPF_10_‐LiPA_25_ protocol, capable of genotyping HPV6, 11, 16, 18, 31, 33, 34, 35, 39, 40, 42, 43, 44, 45, 51, 52, 53, 54, 56, 58, 59, 66, 68, 70, and 74 (version 1; Laboratory Biomedical Products, Rijswijk, the Netherlands). For this study, we selected 711 invasive squamous cell carcinomas showing exclusively the presence of HPV16 DNA after LiPA_25_ genotyping. This data set consisted of samples from the cervix (*n* = 170), vulva (*n* = 128), vagina (*n* = 121), penis (*n* = 119), and anus (*n* = 172), spanning 35 different countries within three geographical regions: Europe, Central/South America, and Asia (Table S1).

Specimens were received anonymously and allocated a unique identification number upon reception, and the respective local and ICO ethic committees approved all the study protocols.

### Identification and selection of the most informative regions

The most variable regions in the HPV16 genome were identified to maximize sequence diversity and phylogenetic signal in the targeted DNA fragments. We retrieved 109 HPV16 complete genome sequences from GenBank. Coding regions were aligned at the amino acid level with Muscle 3.7 19 (http://www.ebi.ac.uk/Tools/msa/muscle/), while the upstream regulatory region (URR) was aligned at nucleotide level. Phylogenetic inference was performed at nucleotide level for each alignment, as well as for the concatenated full‐length aligned genome, under a maximum likelihood framework using RAxML v7.2.8 20 (http://www.exelixis-lab.org/) and the GTR+Γ_4_ as substitution model. Robustness of tree individual nodes was assessed by bootstrap resampling analysis, as determined with the *‐autoMRE* command 21. Using the full‐length genome data and the *–J MR_DROP* command 22, three rogue taxa were identified to show inconsistent positions during bootstrapping and were excluded from further analyses. The final alignment for the full‐length genome comprised 7925 nucleotides and 548 distinct alignment patterns (Fig. S1). The well‐resolved maximum likelihood phylogenetic trees obtained were employed to compute tree‐based pair‐wise genetic distances (nucleotide substitutions per site) for each pair of taxa and for each genomic region analyzed, using the RAxML –*f x* command. Distances were then normalized with respect to the genetic distance between the corresponding taxa for the complete genome.

### PCR and sequencing

DNA was extracted from four 5‐*μ*m paraffin slices by incubation overnight at 56°C with 250‐*μ*L proteinase K buffer (10 mg/mL proteinase K, 50 mmol/L Tris‐HCl, pH 8.0) followed by incubation at 95°C for 8 min to inactivate proteinase K, and stored at −20°C until use. A 1:10 water dilution of this DNA solution was used for downstream processing. PCR primers were designed to target‐specific HPV16 genome regions, so that well‐described linage‐specific polymorphisms were covered by the corresponding amplicons (Table S2). We also used primers previously designed by Larsson and coworkers 23 to span two positions in the *E6* gene that have been thoroughly analyzed in several studies (i.e., nt 131 and 350, reference sequence GenBank: NC_001526). All PCR reaction mixtures contained: 0.125 U/*μ*L AmpliTaq Gold^®^ DNA Polymerase (Life Technologies, Alcobendas, Spain), 2.0 mmol/L MgCl_2_, 0.2 *μ*mol/L deoxynucleotides triphosphate (Life Technologies, Alcobendas, Spain), 0.2 *μ*mol/L forward and reverse primer (Biolegio , Nijmegen, The Netherlands), and 5 *μ*L DNA solution. PCR conditions were 95°C for 10 min; 40 cycles of 30 sec at 94°C, 30 sec at 58°C, 30 sec at 72°C; plus 7‐min final extension at 72°C. PCR products were Sanger‐sequenced at Genoscreen (Lille, France) in both strands using four pairs of primers. (Table S2).

### Phylogenetic analyses

Phylogenetic relationships of the amplified *E6*,* L2*, and LCR short fragments were placed in the global context of HPV16 genetic variability using an Evolutionary Placement Algorithm on RAxML v7.2.8 with the GTR+Γ_4_ model 19, 20. The algorithm provides likelihood weights for placing the partial sequences into the different nodes in the reference tree, in our case based on the pruned full‐length genome alignment described above. Sequences obtained from our samples were incorporated into the reference alignment with MAFFT v7, and their phylogenetic placement was individually inferred with the *‐f v* command in RAxML 21. We integrated the results for all nodes and used 0.7 as a likelihood cutoff value to assign each sample into a specific variant lineage, namely A1‐3, A4, B, C, and D (Table S3, Fig. S1). Using the 0.7 cutoff, 12 samples (1.7%) could only be classified as belonging to the A lineage and were subsequently classified as HPV16_A1‐3 using a 0.6 cutoff value (Table S3).

### Statistical analyses

A generalized linear model (GLM) with a Poisson distribution for count data and a log‐link function was used to analyze the relationship between HPV16 variants prevalence with the two variables of interest: anatomical location and geographical origin. We explored as well the contribution of all double and triple interactions. Significance level was set at *α* value of 0.05. Analyses were performed using R in RStudio v0.98.939 (RStudio, Inc.). To corroborate the GLM results, differences in HPV16 variant distribution stratifying by anatomical location or by geographical origin were statistically assessed by means of Pearson's chi‐square test and of Fisher's test, respectively. Prevalence ratios (PRs) of HPV16 variants among invasive anogenital cancers between Europe and Central/South America or Asia were estimated using Poisson multivariate regression model with robust variance. The different HPV16 variant lineages (i.e., A1–3, A4, and D) were used as dichotomous variables.

Distribution of the polymorphic site T350G within HPV16_A1 variants was assessed by Pearson's chi‐square test when stratified by geographical origin and by Fisher's test when stratified by anatomical location. To assess the possible differential prevalence of the T|G alleles, we estimated the frequency of this polymorphism within HPV16_A1‐3 variants for all anatomical locations for samples from Europe and Central/South America (Table 3). By focusing on the HPV16_A1‐3 variants, we aimed to avoid the possible different epistatic interactions of the T|G alleles with the genetic background of each HPV16 variant, because the *E6* 350 position is also polymorphic T|G in HPV16_B, monomorphic T in HPV16_C, and monomorphic G in HPV16_D 16. Asian cases were excluded from this analysis due to the small number of samples.

Cancer registry data show that cervical cancers are diagnosed earlier than other anogenital cancers associated with HPVs 6, 8, 9, 10. Also, cancers caused by HPV16 are diagnosed earlier than cancers in the same anatomical location caused by other HPVs 4. To disentangle the effects of virus genetics and anatomical location of the lesion on the age at diagnosis, we have followed a top‐down approach, analyzing first age at cancer diagnosis for all HPV‐related anogenital cancers available from our full clinical data set 4, 6, 8, 9, then for all cases exclusively linked to HPV16, and finally for all cases exclusively linked to HPV16_A1‐3 (Fig. 2). For ages at tumor diagnosis, central values were estimated with the median, dispersion was estimated with the median absolute deviation, and differences were assessed by Wilcoxon–Mann–Whitney test. Bonferroni correction for multiple comparisons was used when applicable.

## Results

### Choice of informative regions and sample set description

We identified, in decreasing order, *E4, E5,* LCR*, L2, E2,* and *E6* as the most informative regions in the HPV16 genome to perform phylogenetic inference (Fig. S2). PCRs were designed for each of these six genomic regions, and the LCR, *L2,* and *E6* targets rendered the best results in terms of amplicon quality and suitability for Sanger sequencing, as well as for the number of samples that tested positive. The final sample set comprised three continents (Europe, Central/South America, and Asia) and five anatomical sites (cervix, vulva, vagina, anus, and penis) (Table 1; Table S1). From 711 initially suitable amplicons, we were able to confidently classify 692 (97.3%) as belonging to HPV16_A1‐3, A4, B, C, or D following an evolutionary placement algorithm (Table S3). Only nine samples belonged within the B or C lineages. Given the low numbers for both B and C lineages in our sample set, these sequences were not included in further analyses.

**Table 1:** Anatomical location and geographical distribution of amplified and classified samples

Anatomical location	Europe		Central/South America		Asia		Total amplified	Total classified
	Amplified	Classified	Amplified	Classified	Amplified	Classified		
Cervix	72	70	71	69	27	26	170	165
Vulva	68	68	36	32	24	23	128	123
Vagina	61	60	51	48	10	9	122	117
Penis	74	73	42	40	3	2	119	115
Anal	79	79	72	72	21	21	172	172
Total	354	350	272	261	85	81	711	692

### Geographical origin and anatomical location of the HPV16 variant distribution

The association between HPV16_A1‐3, A4, and D variants (*n* = 683) with anatomical location and geographical origin was assessed using a GLM analysis. The model that fitted best our observations for the complete data set included the predictors “Geographical origin,” “Anatomical location,” and “Variant” (AIC = 225.88; Table S4). All predictors and their two‐by‐two interactions contributed significantly to the model (*P < *0.0001 in all cases), but the triple interaction did not provide additional explanatory power (*P *=* *0.36). The GLM analysis fitted very well our experimental data, as only <1.4% of all variance in HPV16 variant distribution remained unexplained by the model (Table S4). In our data set, 14.1% of the global variability arose from differential coverage of the three geographical regions (*n* = 342 for Europe, *n* = 261 for Central/South America, and *n* = 80 for Asia), and only 1.7% arose from differential coverage of the five anatomical origins analyzed (*n* = 163 for cervix, *n* = 121 for vulva, *n* = 114 for vagina, *n* = 115 for penis, and *n* = 170 for anus). Thus, the GLM approach allowed us to estimate and account for possible biases associated with design asymmetries in our data. We confirmed further the GLM results by estimating prevalence ratios for the different HPV16 variants stratifying by geography (Table 2) and by using a chi‐square test after stratifying for geography and a Fisher's test after stratifying by anatomical location of the samples (Table S5).

**Table 2:** Prevalence ratio (PR) of HPV16 variants between Europe and Central/South America or Asia

Variant	Europe (%)	Central/South America (%)	Asia (%)	Europe vs. Central/South America		Europe vs. Asia	
				PR	95% CI	PR	95% CI
A1‐3	324 (94.7)	225 (86.2)	49 (61.3)	Ref	—	Ref	—
A4	4 (1.2)	1 (0.4)	26 (32.5)	0.49	0.85–2.84	6.60	4.90–8.88
D	14 (4.1)	35 (13.4)	5 (6.2)	1.75	1.43–2.15	2.00	0.90–4.45
Wald's test				*P *<* *0.001		*P *<* *0.001	

**Figure 1:**
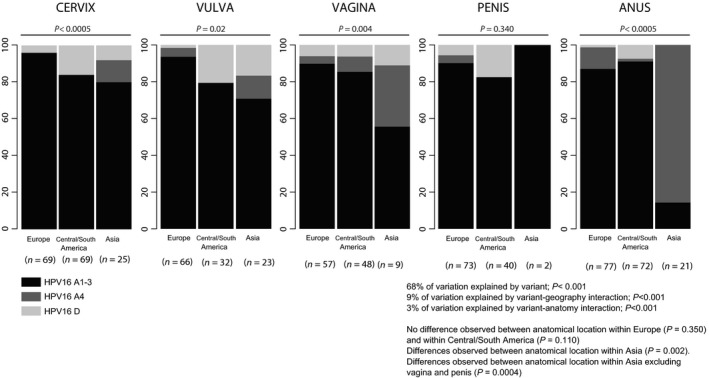
Distribution of HPV16_A1‐3, A4 and D variants depending on geographical regions and anatomical location. For each combination of geography and anatomy, the number of samples is given in parentheses. Values for the contribution of differential variant prevalence (68%), for the contribution of geography (9%), and for the contribution of anatomy (3%) have been generated with a generalized linear model. For each anatomical location, the result of a chi‐square test assessing homogeneity for variant prevalence values between the three geographical origins is provided (e.g., for vaginal cancers, the H0 hypothesis of the variant prevalence values being similar in Europe, Central/South America, and Asia is rejected with *P *=* *0.004). For each geographical origin, the result of a chi‐square test assessing homogeneity for variant prevalence values between the five anatomical locations is provided (e.g., for cancers from Central/South America, the null hypothesis of the variant prevalence values being similar in cervix, vulva, vagina, anus, and penis is accepted with *P *=* *0.074). HPV16, Human papillomavirus type 16.

We estimated that 68.2% of all variation in HPV16 variants abundance corresponded to actual differences in variant prevalence alone (*P < *2.2e^−16^; Table S4). Globally, HPV16_A1‐3 was by far the most prevalent variant, with an overall prevalence of 95% in Europe, 86% in Central/South America, and 61% in Asia (F, Table 2). We quantified further that 9.0% of all variance in variant distribution was explained by differential association of viral lineages with geography (*P < *2.2e^−16^; Table S4). This variation corresponded to a significant 1.7‐fold (95% CI: 1.4–2.1) increase of HPV16_D prevalence in Central/South America and to a significant 6.6‐fold (95% CI: 4.9–8.9) increase of HPV16_A4 prevalence in Asia, in both cases compared with Europe (Table 2, see also Fig. 1). Finally, 2.8% of all variation in variant distribution corresponded to differential association of viral lineages with anatomical location (*P < *2.1e^−05^, Table S4, see also Fig. 1). This variation stemmed from the increased prevalence of HPV16_A4 in vagina and in anus in Asia, where this variant prevailed (Table S5, see also Fig. 1). Differences remained significant even after excluding data from Asia for vagina and penis, both locations with low number of cases (Table S5, see also Fig. 1).

### HPV16 E6 gene T350G polymorphism

Prevalence for the 350G allele within HPV16_A1‐3 ranged between 47% and 59% for Europe and between 59% and 90% for Central/South America. No differences between anatomical locations were observed within each geographical region (respectively, *P *=* *0.617 and *P *=* *0.102 for Europe and Central/South America). However, HPV16_A1‐3 cases from Central/South America showed consistently higher 350G allele frequencies compared with Europe, especially for cervical (*P* = 0.015) and penile (*P* < 0.0005) cancers (Table 3).

**Table 3:** HPV16_A1‐3 variant distribution of the T350G polymorphic site for Europe and Central‐South America and for anatomical location

Anatomical location	Europe (*n* = 277)			Central/South America (*n* = 182)			*F* test^a^
	*N*	350G	%	*N*	350G	%	
Cervix	52	29	55.77	42	34	80.95	0.015
Vulva	49	29	59.18	17	10	58.82	1
Vagina	52	30	57.69	42	29	69.05	0.289
Penis	60	28	46.67	30	27	90.00	<0.0005
Anal	64	38	59.38	51	39	76.47	0.072
*χ*² test^b^	*P *=* *0.617			*P *=* *0.102			

The number of samples (*N*), the samples with 350G allele (350G), and the percentage for the 350G allele frequencies are represented for each anatomical location for Europe and Central/South America. HPV16, Human papillomavirus type 16.

a Within each anatomical location, differences for the 350G allele frequency in the two geographical origins were assessed using Fisher's exact test.

b Within each geographical origin, differences for the 350G allele frequency in the different anatomical locations were assessed using chi‐square test.

### Age at cancer diagnosis

**Figure 2:**
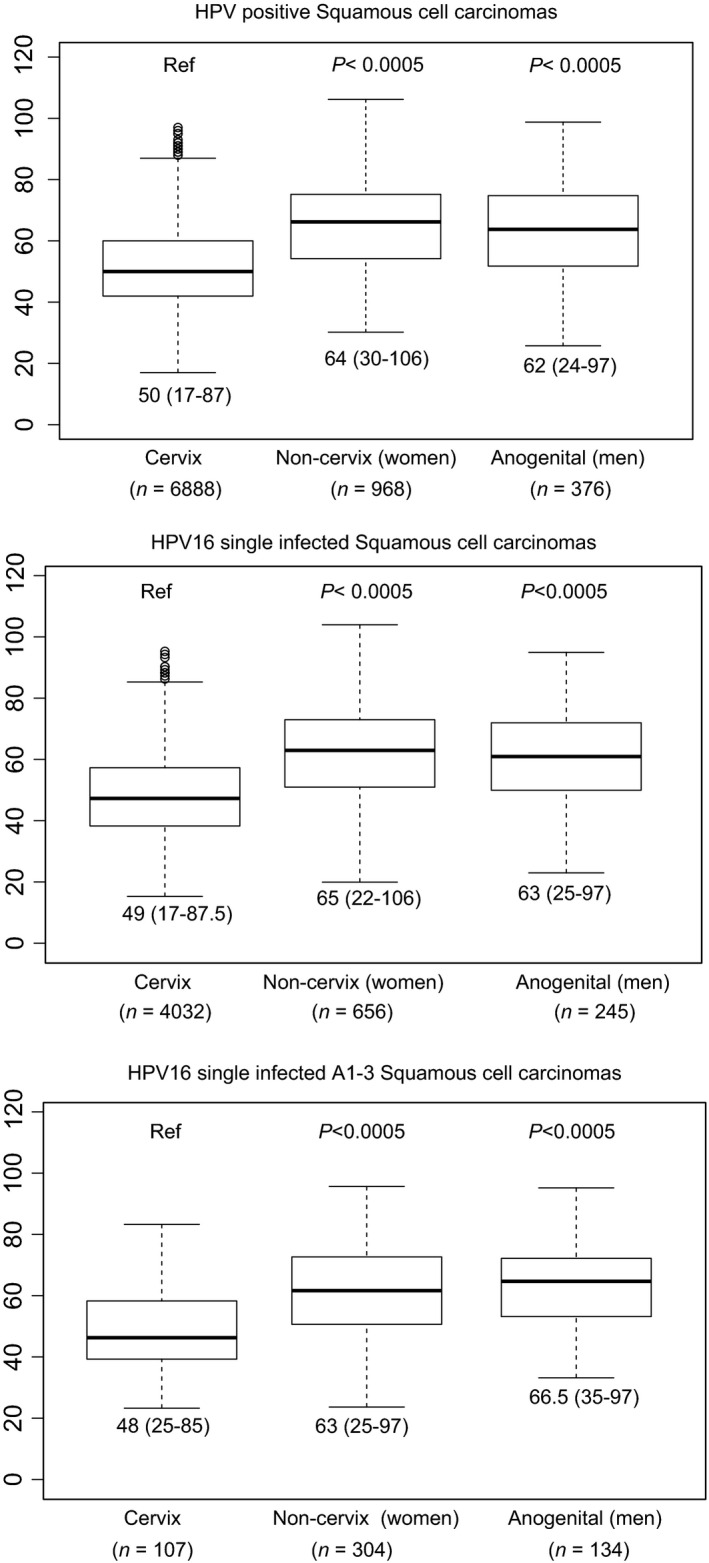
Age at tumor diagnosis for HPV‐positive, HPV16 single infected, and HPV16_A1‐3 single infected invasive SCC stratified by cervix, women anogenital noncervix (encompassing vagina, vulva, and anus), and men anogenital (encompassing anus and penis) samples. For each data set, the bar represents the median, the box encompasses the 25–75% percentiles, and the whiskers encompass the 95% percentiles. Numbers below each graph indicate the median and the range (1.5 × interquartile). Numbers in parentheses indicate the sample size for each location. Values for HPV‐positive and HPV16‐positive SCCs have been taken from data sets published by de Sanjosé et al. (cervix) 4, Sanjose et al. (vulva) 9, Alemany et al. (penis) 6, Alemany et al. (anus) 8, and Alemany et al. (vagina) 10. HPV, human papillomaviruses; SCC, squamous cell carcinomas.

Cervical cancers showed significantly younger ages at diagnosis compared with other anogenital cancers (early fifties vs. early sixties, *P* < 0.0005) regardless of the oncogenic HPV type or of the HPV16 variant driving the cancer (Fig. 2, see also Table S6). Notably, no significant differences were observed for age at cancer diagnosis among noncervical cancers (Fig. S3, see also Table S7).

## Discussion

In this study, we have assessed the HPV16 variant diversity in a comprehensive set of invasive tumors of the cervix, vulva, vagina, penis, and anus, analyzing the HPV16 variant distribution in 692 invasive squamous cancer samples from Europe, Central/South America, and Asia.

We have quantified for the first time the relative contributions of variant differential abundance, geographical origin, and anatomical location of the anogenital cancers to the observation of differential prevalence distribution of HPV16 variants. Our results show that there are no large differences between HPV16 lineage prevalence values among the anogenital cancers. The most prevalent viral lineage was by far HPV16_A1‐3, independently of geographical origin and anatomical location of the samples, with the only exception of anal cancers in Asia, dominated by HPV16_A4, albeit based on small numbers. We have further estimated the contribution of geography and anatomical location to the observed differential HPV16 variant prevalence. The geographical origin of the cancer sample explains roughly 9% of all diversity in viral lineage distribution, and this contribution arises essentially from the increased prevalence of HPV16_A4 in samples from Asia and of HPV16_D in samples from Central/South America. In contrast, the anatomical location of the anogenital cancer explains only <3% of the observed diversity in viral lineages distribution. Indeed, we have not observed significant prevalence differences for HPV16 variants between anatomical locations of the anogenital cancers within Europe or Central/South America. In Asia, however, the higher contribution of HPV16_A4 variant in anogenital cancers exhibited a significant prevalence peak for anal cancers.

Variant distribution and diversity in HPV16 have mainly focused on the uterus cervix 16, 24, 25, but a sound description for viral lineages in other anogenital cancers sites was still wanting. Here, we have characterized the HPV16 variant component in a total of 692 anogenital invasive squamous cancers, including more than 550 cases from the vulva, vagina, penis, or anus. Our results confirm previous results with cervical samples and show further that the repertoire of viral HPV16 variants in anogenital cancers is largely the same regardless of the anatomical location. Consistent with our observations, two previous small studies in Northern Europe (HPV positive total *N* = 40; HPV16 positive *N* = 31) and in North America (HPV positive total *N* = 14; HPV16 positive *N* = 9) also reported an increased prevalence of HPV16_A1‐3 variants in vulvar cancer (*N* = 29/31; *N* = 5/9 respectively) 26, 27. Regarding vaginal cancer, the only previous study analyzing HPV16 variants (HPV positive total *N* = 37; HPV16 positive total *N* = 26) showed exclusively the presence of HPV16_A1‐3 variants in European samples 26. For anal cancer, a Canadian study (HPV positive total *N* = 96; HPV16 positive total *N* = 79) reported around 90% prevalence for HPV16_A variants 28. Finally, and concerning viral diversity in HPV16‐associated penile cancers, an Italian study (HPV positive total *N* = 19; HPV16 positive total *N* = 18) showed above 40% prevalence for both HPV16_A1‐3 and D variants, along with above 10% minor nonnegligible contribution of HPV16_B variants 29. However, a Mexican series of penile cancer samples (HPV positive total *N* = 67; HPV16 positive total *N* = 57) showed 92% prevalence of HPV16_A1‐3 and 8% prevalence of HPV16_D 30. In certain cases, the use in previous literature of imprecise naming schemes for HPV16 variants hampers a proper comparison. To avoid ambiguity, we have adhered here to the HPV16 variant terminology as standardized by Burk and coworkers 15 and strongly encourage further research on HPV variants to stick to it. Previous HPV16 variant nomenclatures included potentially misleading geographical references (e.g., “European”) or ill‐defined arbitrary classifications (e.g., “prototype” or “nonprototype”). The use of a geography‐based nomenclature conveys a message of a close match between differential HPV16 variants prevalence and geography, which is not justified neither by the best previously available data 16 nor by our results presented here.

Minor variations in the viral genome may be responsible for important changes regarding increased persistence or viral load 36, 37. In addition, the adaptive host–pathogen interaction may further condition the differential probability for clearance or for eventual malignization of HPV16 infections 35, 36. To tackle this connection between viral genotypic diversity and cancer risk, a long‐studied candidate has been the T350G(L83V) single‐nucleotide polymorphism in the HPV16 genome 38, 39. In vitro studies have suggested an increased transformation potential for the 83V allele, especially for the HPV16_D lineage 38, 40, although these results may be linked to a specific host genetic background 39. In European populations, prospective studies in cervical lesions as well as case–control studies have also communicated inconsistent results regarding the involvement of the T350G polymorphism in the persistence and progression to cancer 17, 18, 33, 41. To address the question of the differential HPV16_*E6* 350G allele frequencies as a function of the geographical origin and the anatomical location of the cases, we focused exclusively on HPV16_A1‐3 samples from Europe and Central/South America. We found that the 350G allele frequency did not significantly differ between anatomical locations for samples from the same geographical origin. In addition, our analysis revealed an increase in 350G allele frequency in samples from Central/South America compared with samples from Europe, consistent for all anatomical locations except for the vulva. This trend is in agreement with previous studies reporting an increased frequency of the 350G allele in Central/South America compared with European populations 31, 32, 33, 34, as well as with the minor contribution of this allele in vulvar and in vaginal lesions 23, 26.

Finally, we estimated the possible influence of the HPV16 variant on the age at cancer diagnosis. The rationale behind is threefold. First, cancer registry data show that cervical cancers are diagnosed earlier than other anogenital cancers associated with HPVs (www.hpvcentre.net). Second the studies from our group also show that cervical cancers caused by more aggressive HPVs, such as HPV16, HPV18, or HPV45, are diagnosed earlier than cervical cancers caused by other HPVs 4. Third, the relative contribution of the different HPVs varies depending on the anatomical location of the cancer. Thus, the observed differences in age at diagnosis in HPV‐related cancers of different anatomical origin could be linked to specific characteristics of the target tissue and/or to the different prevalence of the underlying viral agents. Making a coherent picture out of all available facts remains, however, a conundrum, because the contribution of HPV16 in noncervical cancers is higher than in cervical cancer: 61% in cervix 4, 62.9% in penis 6, 72.5% in vulva 9, and 75.8% in anus 8. The only exception to this trend is vaginal cancers, showing a 57% contribution of HPV16 10. One would thus expect that the increased contribution of HPV16 in noncervical cancers would result in earlier age at diagnosis when comparing HPV‐related cancers among locations, but this is not the case. Our study design offered a unique opportunity to disentangle both alternatives and to test these hypotheses, as we have gathered a large sample set of HPV16‐monoinfected cancers of five different anatomical origins. One explanation could have been that HPV16 variants show different prevalence in cancers from different anatomical locations, but the results communicated here suggest that such differences are minor. Remarkably, our results for the complete series on HPVs and anogenital cancers 4, 6, 8, 9, 10 show that differences in age at diagnosis remain unchanged between cervical cancers (diagnosed in the early fifties) and noncervical cancers (diagnosed in the early sixties) when only HPV16 monoinfections are considered, and even after focusing on cancers associated to a single viral lineage, namely the most prevalent HPV16_A1‐3. We propose that tissue‐specific characteristics of the transitional epithelia at the uterus cervix may underlie the observed trend in age at diagnosis, reflecting an enhanced propensity to infection and/or a lower rate of infection clearing, resulting in an increased vulnerability to HPV16‐driven cervical carcinogenesis 42. The anatomy of anal mucosa encompasses also the transitional epithelia of a squamous‐columnar junction, but nevertheless cervical and anal cancers largely differ in incidence and prevalence, age at diagnosis as well as in the repertoire of HPVs and in the contribution of HPV16 to malignization 4, 8. A particular cellular population in the squamous‐columnar junction of the uterus cervix, the so‐called cuboidal cells, which retain phenotypic features of embryonic stem cells, is enriched among transformed cells located in the uterus cervix, suggesting that they are implicated in cervical neoplasia after persistent HPVs infection 43. However, the microanatomy of the anal transformation zone is not identical to that of cervical transformation zone: cells in the cervical squamous‐columnar junction are monolayered, they are in direct contact with the basal membrane, and they display a immunophenotype different tumor 44. Such histochemical differences between cervical and anal transformation zones most likely underlie the large epidemiological differences between cervical and anal HPV‐induced cancers in terms of incidence, prevalence, and age at diagnosis.

Despite the large sample size and the sound molecular identification of viral variants, our study suffers from a number of limitations. First, we chose to restrict ourselves to a very homogeneous study subject, namely invasive squamous cell carcinomas containing exclusively HPV16 DNA. By doing so, we could only recover in our repository enough samples from Europe, Central/South America, and Asia, as we did not have access to good quality samples from the African continent. Indeed, a thorough study on the evolution of any human pathogen should aim to sample the host–pathogen interaction there where the genetic diversity of the host is largest, that is, Africa, for humans 45. Second, we recognize that given the variant variability observed in anal samples, the study would have benefited from an increase in samples collected from Asia. Third, due to the nature of our study, we did not have access to any data regarding patient ethnicity and we were thus constrained to proxy the genetic background of the patient after the geographical origin of the sample. Although the geographical origin of the clinical sample is shown to rather reliably reflect the ethnic origin of the patient, even in admixed populations such as in Central/South America 46, it would have been desirable to generate the genetic data from both human and virus from the same infection. Finally, we have used the broad geographical units defined by the United Nations to stratify geography, but we are nevertheless aware that these units cover large differences in human genetic diversity that may thus result flattened.

In conclusion, our results provide for the first time a sound estimate of the differential contribution of HPV16 variants to anogenital cancers in Europe, Central/South America, and Asia. We identify geographical origin and anatomical location as minor factors for explaining the differential prevalence of HPV16 variants in anogenital cancers. Instead, our results suggest that the increased prevalence of HPV16_A1‐3 may reflect a genuinely enhanced fitness for this variant to cause cancer. Only a large prospective series exploring HPV16 variants prevalence on clinically asymptomatic infections, monitoring the initial steps of viral colonization of anogenital mucosas and following differential viral persistence and accounting for the patient's genetic background, will ultimately provide answers about the extent of the differential fitness of these viral lineages and will help understand the host interplay of the most oncogenic HPV.

## Conflict of Interest

None declared.

## Supporting information


**Figure S1.** Cumulative pair‐wise distance frequencies for HPV16 genes and control region: Pair‐wise distances (substitutions per site) are calculated for the full‐genome, LCR, and all ORFs of reference sequences. Horizontal plain gray line represents the pair‐wise distance 95th percentile. E2 – E4 (E2 minus E4) stands for the E2 gene nonoverlapping with the E4 gene.

**Figure S2.** Mid‐point rooted HPV16 best‐known maximum likelihood phylogenetic tree, constructed using 109 unique full‐length genome sequences. HPV16 lineages are classified into four variants: A, B, C, and D. Bootstrap values above 700 are displayed closed to the corresponding node. GenBank accession numbers are given for all entries.

**Figure S3.** Age at tumor diagnosis for HPV positive, HPV16 single‐infected band HPV16 A1‐3 invasive squamous cell carcinomas stratified by anatomical location: Box plots represent the median, the 25% and 75% quantiles. Median and range (1.5 × interquantile) are represented in brackets below the box plots. Number of samples is represented in brackets below the anatomical location. HPV16‐positive samples include all the types detectable through SPF10‐LIPA25 protocol (version 1; Laboratory Biomedical Products, Rijswijk, the Netherlands).

**Table S1.** Sample distribution per anatomical location, geographical region, and country.
**Table S2**. Primer design.
**Table S3.** Likelihood weights for the attribution of each individual sequence to each (sub) variant, number of samples, and percentage.
**Table S4.** Generalized linear model (GLM) and analysis of deviance.
**Table S5.** HPV16 A1‐3, A4 and D variant distribution by anatomical location within each geographical area.
**Table S6.** Age at tumor diagnosis for invasive squamous invasive carcinomas HPV positive, HPV negative, HPV16 single infected, and HPV16 A1‐A2‐A3 stratified by cervix, noncervix (women), and anogenital (men) samples.
**Table S7.** Age at tumor diagnosis for invasive squamous invasive carcinomas HPV positive, HPV16 single infected, and HPV16 A1‐A2‐A3 stratified by anatomical location.
**Table S8.** Collaborating centers at the RIS HPV TT and HPV VVAP study groups.

## References

[1] IARC . 2012 IARC monographs on the evaluation of carcinogenic risks to humans ‐ biological agents. 100B:100B.

[2] Munoz, N. , F. X. Bosch , S. de Sanjose , R. Herrero , X. Castellsague , K. V. Shah , et al. 2003 Epidemiologic classification of human papillomavirus types associated with cervical cancer. N. Engl. J. Med. 348:518–527. 12571259 10.1056/NEJMoa021641

[3] Walboomers, J. M. , M. V. Jacobs , M. M. Manos , F. X. Bosch , J. A. Kummer , K. V. Shah , et al. 1999 Human papillomavirus is a necessary cause of invasive cervical cancer worldwide. J. Pathol. 189:12–19. 10451482 10.1002/(SICI)1096-9896(199909)189:1<12::AID-PATH431>3.0.CO;2-F

[4] de Sanjose, S. , W. G. Quint , L. Alemany , D. T. Geraets , J. E. Klaustermeier , B. Lloveras , et al. 2010 Human papillomavirus genotype attribution in invasive cervical cancer: a retrospective cross‐sectional worldwide study. Lancet Oncol. 11:1048–1056. 20952254 10.1016/S1470-2045(10)70230-8

[5] De Vuyst, H. , G. M. Clifford , M. C. Nascimento , M. M. Madeleine , and S. Franceschi . 2009 Prevalence and type distribution of human papillomavirus in carcinoma and intraepithelial neoplasia of the vulva, vagina and anus: a meta‐analysis. Int. J. Cancer 124:1626–1636. 19115209 10.1002/ijc.24116

[6] Alemany, L. , A. Cubilla , G. Halec , E. Kasamatsu , B. Quirós , E. Masferrer , et al. 2016 Role of human papillomavirus in penile carcinomas Worldwide. Eur. Urol. 10.1016/j.eururo.2015.12.007 26762611

[7] Giuliano, A. R. , G. Tortolero‐Luna , E. Ferrer , A. N. Burchell , S. de Sanjose , S. K. Kjaer , et al. 2008 Epidemiology of human papillomavirus infection in men, cancers other than cervical and benign conditions. Vaccine 26(Suppl 1):K17–K28. 18847554 10.1016/j.vaccine.2008.06.021 PMC4366004

[8] Alemany, L. , M. Saunier , I. Alvarado‐Cabrero , B. Quirós , J. Salmeron , H.‐R. Shin , et al. 2015 Human papillomavirus DNA prevalence and type distribution in anal carcinomas worldwide. Int. J. Cancer 136:98–107. 24817381 10.1002/ijc.28963 PMC4270372

[9] de Sanjosé, S. , L. Alemany , J. Ordi , S. Tous , M. Alejo , S. M. Bigby , et al. 2013 Worldwide human papillomavirus genotype attribution in over 2000 cases of intraepithelial and invasive lesions of the vulva. Eur. J. Cancer 49:3450–3461. 23886586 10.1016/j.ejca.2013.06.033

[10] Alemany, L. , M. Saunier , L. Tinoco , B. Quirós , I. Alvarado‐Cabrero , M. Alejo , et al. 2014 Large contribution of human papillomavirus in vaginal neoplastic lesions: a worldwide study in 597 samples. Eur. J. Cancer 50:2846–2854. 25155250 10.1016/j.ejca.2014.07.018

[11] Bruni, L. , M. Diaz , X. Castellsague , E. Ferrer , F. X. Bosch , and S. de Sanjose . 2010 Cervical human papillomavirus prevalence in 5 continents: meta‐analysis of 1 million women with normal cytological findings. J. Infect. Dis. 202:1789–1799. 21067372 10.1086/657321

[12] Bzhalava, D. , P. Guan , S. Franceschi , J. Dillner , and G. Clifford . 2013 A systematic review of the prevalence of mucosal and cutaneous human papillomavirus types. Virology 445:224–231. 23928291 10.1016/j.virol.2013.07.015

[13] Bravo, I. G. , and M. Félez‐Sánchez . 2015 Papillomaviruses: viral evolution, cancer and evolutionary medicine. Evol. Med. Public Health 2015:32–51. 25634317 10.1093/emph/eov003 PMC4356112

[14] de Villiers, E. M. , C. Fauquet , T. R. Broker , H. U. Bernard , and H. Zur Hausen . 2004 Classification of papillomaviruses. Virology 324:17–27. 15183049 10.1016/j.virol.2004.03.033

[15] Burk, R. D. , A. Harari , and Z. Chen . 2013 Human papillomavirus genome variants. Virology 445:232–243. 23998342 10.1016/j.virol.2013.07.018 PMC3979972

[16] Cornet, I. , T. Gheit , S. Franceschi , J. Vignat , R. D. Burk , B. S. Sylla , et al. 2012 Human papillomavirus type 16 genetic variants: phylogeny and classification based on E6 and LCR. J. Virol. 86:6855–6861. 22491459 10.1128/JVI.00483-12 PMC3393538

[17] Grodzki, M. , G. Besson , C. Clavel , A. Arslan , S. Franceschi , P. Birembaut , et al. 2006 Increased risk for cervical disease progression of French women infected with the human papillomavirus type 16 E6‐350G variant. Cancer Epidemiol. Biomarkers Prev. 15:820–822. 16614130 10.1158/1055-9965.EPI-05-0864

[18] Gheit, T. , I. Cornet , G. M. Clifford , T. Iftner , C. Munk , M. Tommasino , et al. 2011 Risks for persistence and progression by human papillomavirus type 16 variant lineages among a population‐based sample of Danish women. Cancer Epidemiol. Biomarkers Prev. 20:1315–1321. 21527576 10.1158/1055-9965.EPI-10-1187

[19] Stark, M. , S. A. Berger , A. Stamatakis , and C. von Mering . 2010 MLTreeMap–accurate maximum likelihood placement of environmental DNA sequences into taxonomic and functional reference phylogenies. BMC Genom. 11:461. 10.1186/1471-2164-11-461 PMC3091657 20687950

[20] Berger, S. A. , and A. Stamatakis . 2011 Aligning short reads to reference alignments and trees. Bioinformatics 27:2068–2075. 21636595 10.1093/bioinformatics/btr320

[21] Stamatakis, A. 2006 RAxML‐VI‐HPC: maximum likelihood‐based phylogenetic analyses with thousands of taxa and mixed models. Bioinformatics 22:2688–2690. 16928733 10.1093/bioinformatics/btl446

[22] Pattengale, N. D. , A. J. Aberer , K. M. Swenson , A. Stamatakis , and B. M. E. Moret . 2011 Uncovering hidden phylogenetic consensus in large data sets. IEEE/ACM Trans Comput Biol Bioinform [Internet] 8:902–911. Available from: http://www.ncbi.nlm.nih.gov/pubmed/21301032 (accessed: September 30, 2015) 10.1109/TCBB.2011.28 21301032

[23] Larsson, G. L. , G. Helenius , S. Andersson , F. Elgh , B. Sorbe , and M. G. Karlsson . 2012 Human papillomavirus (HPV) and HPV 16‐variant distribution in vulvar squamous cell carcinoma in Sweden. Int. J. Gynecol. Cancer 22:1413–1419. 23013732 10.1097/IGC.0b013e31826a0471

[24] Yamada, T. , C. M. Wheeler , A. L. Halpern , A. C. Stewart , A. Hildesheim , and S. A. Jenison . 1995 Human papillomavirus type 16 variant lineages in United States populations characterized by nucleotide sequence analysis of the E6, L2, and L1 coding segments. J. Virol. 69:7743–7753. 7494284 10.1128/jvi.69.12.7743-7753.1995 PMC189716

[25] Ho, L. , S. Y. Chan , V. Chow , T. Chong , S. K. Tay , L. L. Villa , et al. 1991 Sequence variants of human papillomavirus type 16 in clinical samples permit verification and extension of epidemiological studies and construction of a phylogenetic tree. J. Clin. Microbiol. 29:1765–1772. 1663516 10.1128/jcm.29.9.1765-1772.1991 PMC270207

[26] Larsson, G. L. , G. Helenius , S. Andersson , B. Sorbe , and M. G. Karlsson . 2013 Prognostic impact of human papilloma virus (HPV) genotyping and HPV‐16 subtyping in vaginal carcinoma. Gynecol. Oncol. 129:406–411. 23402906 10.1016/j.ygyno.2013.02.004

[27] de Koning, M. N. C. , W. G. V. Quint , and E. C. Pirog . 2008 Prevalence of mucosal and cutaneous human papillomaviruses in different histologic subtypes of vulvar carcinoma. Mod. Pathol. 21:334–344. 18192968 10.1038/modpathol.3801009

[28] Ouhoummane, N. , M. Steben , F. Coutlée , T. Vuong , P. Forest , C. Rodier , et al. 2013 Squamous anal cancer: patient characteristics and HPV type distribution. Cancer Epidemiol. 37:807–812. 24139594 10.1016/j.canep.2013.09.015

[29] Tornesello, M. L. , M. L. Duraturo , S. Losito , G. Botti , S. Pilotti , B. Stefanon , et al. 2008 Human papillomavirus genotypes and HPV16 variants in penile carcinoma. Int. J. Cancer 122:132–137. 17764110 10.1002/ijc.23062

[30] López‐Romero, R. , C. Iglesias‐Chiesa , B. Alatorre , K. Vázquez , P. Piña‐Sánchez , I. Alvarado , et al. 2013 HPV frequency in penile carcinoma of Mexican patients: important contribution of HPV16 European variant. Int. J. Clin. Exp. Pathol. 6:1409–1415. 23826423 PMC3693207

[31] Villa, L. L. , L. Sichero , P. Rahal , O. Caballero , A. Ferenczy , T. Rohan , et al. 2000 Molecular variants of human papillomavirus types 16 and 18 preferentially associated with cervical neoplasia. J. Gen. Virol. 81:2959–2968. 11086127 10.1099/0022-1317-81-12-2959

[32] Zuna, R. E. , E. Tuller , N. Wentzensen , C. Mathews , R. A. Allen , R. Shanesmith , et al. 2011 HPV16 variant lineage, clinical stage, and survival in women with invasive cervical cancer. Infect. Agent Cancer 6:19. 22035468 10.1186/1750-9378-6-19 PMC3226431

[33] Cornet, I. , T. Gheit , M. R. Iannacone , J. Vignat , B. S. Sylla , A. Del Mistro , et al. 2013 HPV16 genetic variation and the development of cervical cancer worldwide. Br. J. Cancer 108:240–244. 23169278 10.1038/bjc.2012.508 PMC3553516

[34] Freitas, L. B. , Z. Chen , E. F. Muqui , N. A. T. Boldrini , A. E. Miranda , L. C. Spano , et al. 2014 Human papillomavirus 16 non‐European variants are preferentially associated with high‐grade cervical lesions. PLoS ONE 9:e100746. 24983739 10.1371/journal.pone.0100746 PMC4077691

[35] Lopera, E. A. , A. Baena , V. Florez , J. Montiel , C. Duque , T. Ramirez , et al. 2014 Unexpected inverse correlation between Native American ancestry and Asian American variants of HPV16 in admixed Colombian cervical cancer cases. Infect. Genet. Evol. 28:339–348. 25446942 10.1016/j.meegid.2014.10.014

[36] Banister, C. E. , A. R. Messersmith , B. Cai , L. B. Spiryda , S. H. Glover , L. Pirisi , et al. 2015 Disparity in the persistence of high‐risk human papillomavirus genotypes between African American and European American women of college age. J. Infect. Dis. 211:100–108. 25028692 10.1093/infdis/jiu394 PMC4326315

[37] Amador‐Molina, A. , J. L. González‐Montoya , A. García‐Carrancá , A. Mohar , and M. Lizano . 2013 Intratypic changes of the E1 gene and the long control region affect ori function of human papillomavirus type 18 variants. J. Gen. Virol. 94:393–402. 23100366 10.1099/vir.0.045807-0

[38] Zehbe, I. , C. Richard , C. A. DeCarlo , A. Shai , P. F. Lambert , H. Lichtig , et al. 2009 Human papillomavirus 16 E6 variants differ in their dysregulation of human keratinocyte differentiation and apoptosis. Virology 383:69–77. 18986660 10.1016/j.virol.2008.09.036 PMC2945146

[39] Zehbe, I. , H. Lichtig , A. Westerback , P. F. Lambert , M. Tommasino , and L. Sherman . 2011 Rare human papillomavirus 16 E6 variants reveal significant oncogenic potential. Mol. Cancer. 10:77. 21702904 10.1186/1476-4598-10-77 PMC3144020

[40] Zehbe, I. , E. Wilander , H. Delius , and M. Tommasino . 1998 Human papillomavirus 16 E6 variants are more prevalent in invasive cervical carcinoma than the prototype. Cancer Res. 58:829–833. 9485042

[41] Tornesello, M. L. , S. Losito , G. Benincasa , F. Fulciniti , G. Botti , S. Greggi , et al. 2011 Human papillomavirus (HPV) genotypes and HPV16 variants and risk of adenocarcinoma and squamous cell carcinoma of the cervix. Gynecol. Oncol. 121:32–42. 21211829 10.1016/j.ygyno.2010.12.005

[42] Crum, C. P. 2000 Contemporary theories of cervical carcinogenesis: the virus, the host, and the stem cell. Mod. Pathol. 13:243–251. 10757335 10.1038/modpathol.3880045

[43] Herfs, M. , Y. Yamamoto , A. Laury , X. Wang , M. R. Nucci , M. E. McLaughlin‐Drubin , et al. 2012 A discrete population of squamocolumnar junction cells implicated in the pathogenesis of cervical cancer. Proc. Natl Acad. Sci. USA 109:10516–10521. 22689991 10.1073/pnas.1202684109 PMC3387104

[44] Yang, E. J. , M. C. Quick , S. Hanamornroongruang , K. Lai , L. A. Doyle , F. D. McKeon , et al. 2015 Microanatomy of the cervical and anorectal squamocolumnar junctions: a proposed model for anatomical differences in HPV‐related cancer risk. Mod. Pathol. 28:994–1000. Available from: http://dx.doi.org/10.1038/modpathol.2015.54 (accessed 27 September 2015) 25975286 10.1038/modpathol.2015.54 PMC4490106

[45] The 1000 genomes project consortium .2012 An integrated map of genetic variation from 1092 human genomes. Nature 491:56–65. 23128226 10.1038/nature11632 PMC3498066

[46] Homburger, J. R. , A. Moreno‐Estrada , C. R. Gignoux , D. Nelson , E. Sanchez , P. Ortiz‐Tello , et al. 2015 Genomic insights into the ancestry and demographic history of South America. PLoS Genet. 11:e1005602. 26636962 10.1371/journal.pgen.1005602 PMC4670080

