# Supplementary material for: HPV16 variants distribution in invasive cancers of the cervix, vulva, vagina, penis, and anus
Source: Cancer Med. 2016 Sep 21;5(10):2909–19. doi: 10.1002/cam4.870 (PMC5083745; doi:10.1002/cam4.870)
Supplement: Supplementary file 1 — Figure S1. Cumulative pair‐wise distance frequencies for HPV16 genes and control region: Pair‐wise distances (substitutions per site) are calculated for the full‐genome, LCR, and all ORFs of reference sequences. Horizontal plain gray line represents the pair‐wise distance 95th percentile. E2 – E4 (E2 minus E4) stands for the E2 gene nonoverlapping with the E4 gene. [file CAM4-5-2909-s001.pdf]

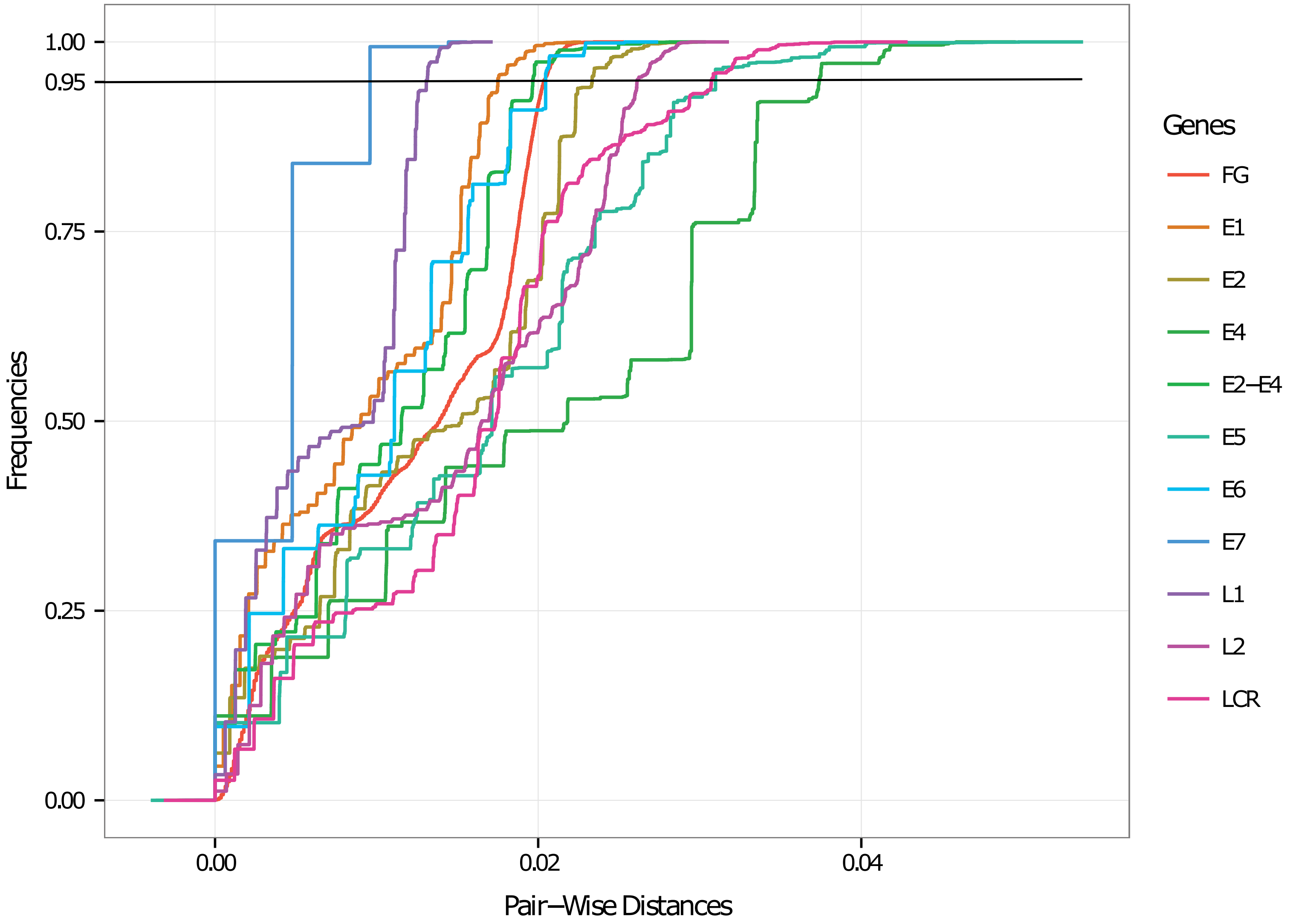

Figure S1: Cumulative pair-wise distance frequencies for HPV16 genes and control region: Pair-wise distances (substitutions per site) are calculated for the full-genome, LCR and all ORFs of reference sequences.Horitzontal plain grey line represent the pair-wise distance 95th percentile. E2-E4 (E2 minus E4) stands for the E2 gene non-overlapping with the E4 gene.
