# Supplementary material for: HPV16 variants distribution in invasive cancers of the cervix, vulva, vagina, penis, and anus
Source: Cancer Med. 2016 Sep 21;5(10):2909–19. doi: 10.1002/cam4.870 (PMC5083745; doi:10.1002/cam4.870)
Supplement: Supplementary file 3 — Figure S3. Age at tumor diagnosis for HPV positive, HPV16 single‐infected band HPV16 A1‐3 invasive squamous cell carcinomas stratified by anatomical location: Box plots represent the median, the 25% and 75% quantiles. Median and range (1.5 × interquantile) are represented in brackets below the box plots. Number of samples is represented in brackets below the anatomical location. HPV16‐positive samples include all the types detectable through SPF10‐LIPA25 protocol (version 1; Laboratory Biomedical Products, Rijswijk, the Netherlands). [file CAM4-5-2909-s003.pdf]

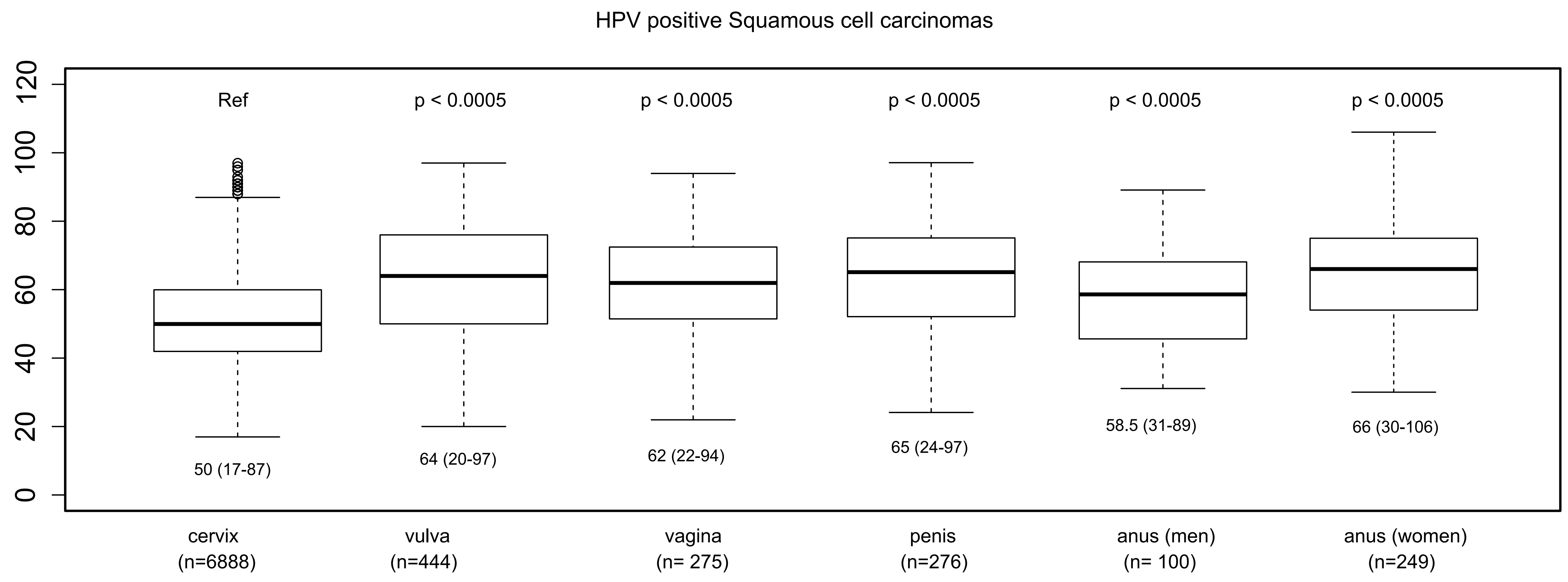

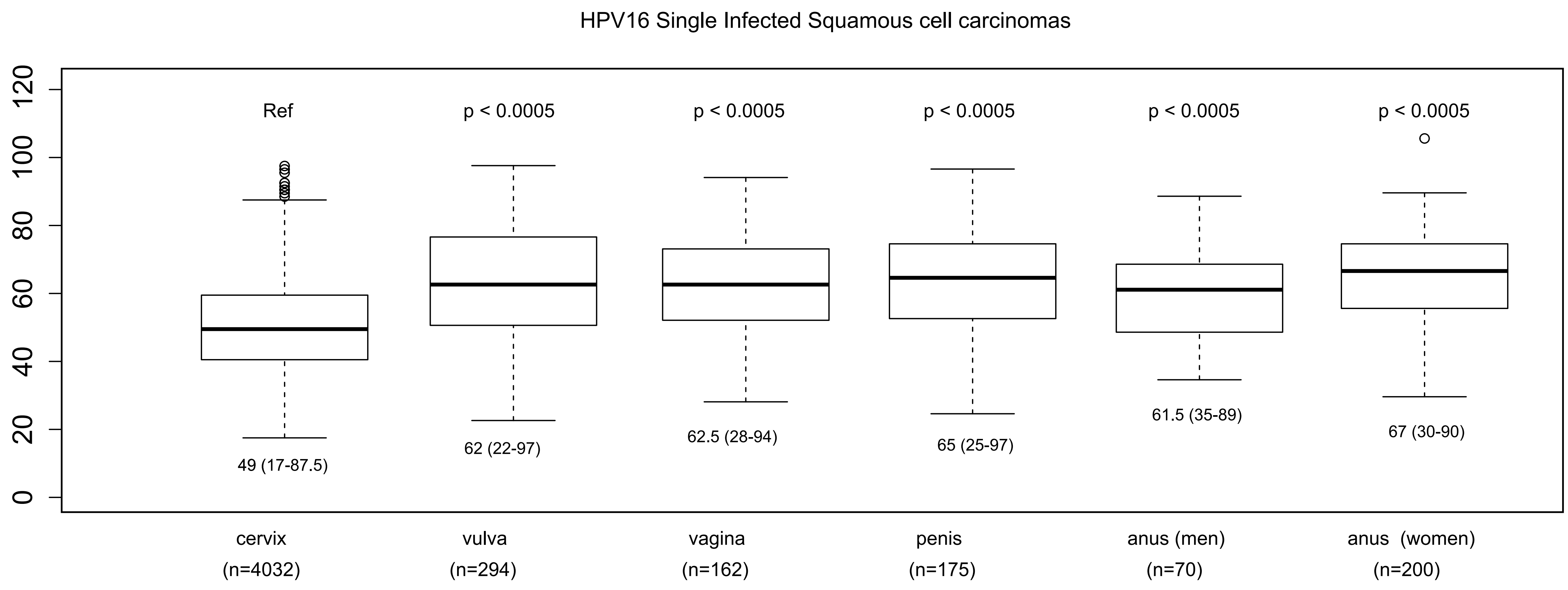

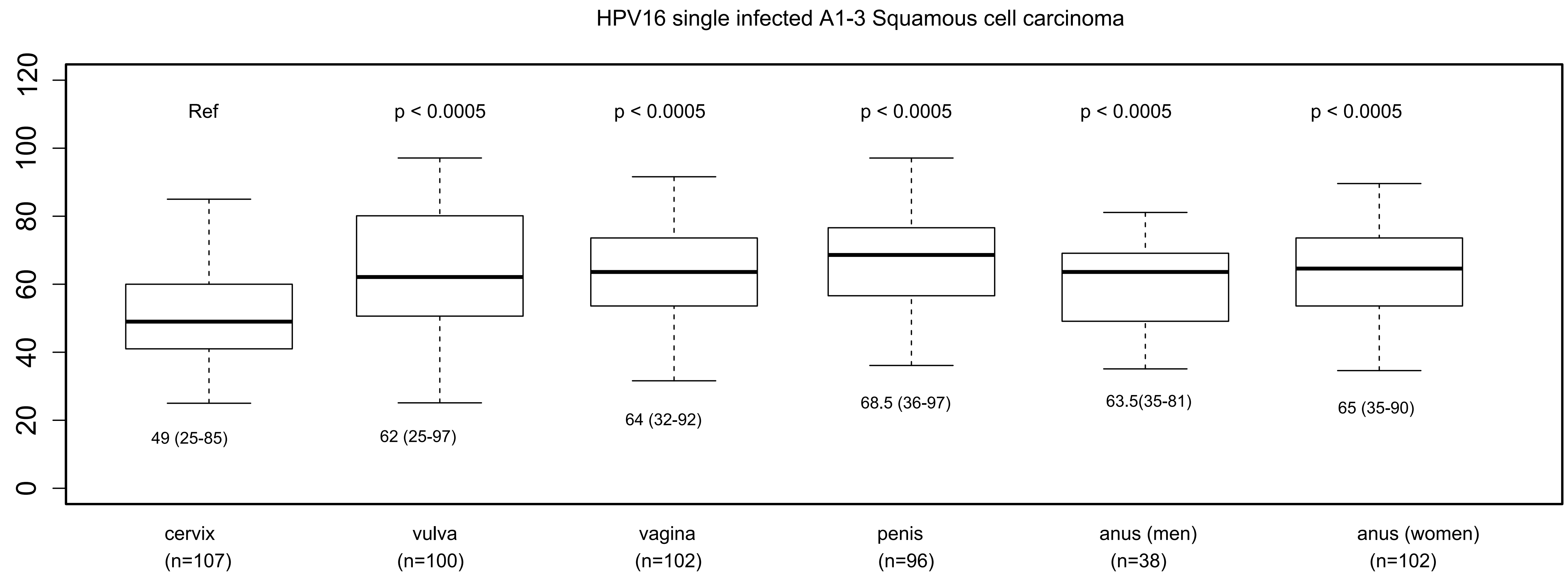

Figure S3: Age at tumour diagnosis for HPV positive, HPV16 single infected and HPV16 A1-3 invasive squamous cell carcinomas stratified by anatomical location: Boxplots represent the median, the 25% and 75% quantiles. Median and range (1.5 x Inter-quantile) are represented in brackets below the boxplots. Number of samples are represented in brackets below the anatomical location. HPV16 positive samples include all the types detectable through SPF10-LIPA25 protocol (version 1; Laboratory Biomedical Products, Rijswijk, Netherlands)
