# Supplementary material for: HPV16 variants distribution in invasive cancers of the cervix, vulva, vagina, penis, and anus
Source: Cancer Med. 2016 Sep 21;5(10):2909–19. doi: 10.1002/cam4.870 (PMC5083745; doi:10.1002/cam4.870)
Supplement: Supplementary file 4 — Table S1. Sample distribution per anatomical location, geographical region, and country. Table S2. Primer design. Table S3. Likelihood weights for the attribution of each individual sequence to each (sub) variant, number of samples, and percentage. Table S4. Generalized linear model (GLM) and analysis of deviance. Table S5. HPV16 A1‐3, A4 and D variant distribution by anatomical location within each geographical area. Table S6. Age at tumor diagnosis for invasive squamous invasive carcinomas HPV positive, HPV negative, HPV16 single infected, and HPV16 A1‐A2‐A3 stratified by cervix, noncervix (women), and anogenital (men) samples. Table S7. Age at tumor diagnosis for invasive squamous invasive carcinomas HPV positive, HPV16 single infected, and HPV16 A1‐A2‐A3 stratified by anatomical location. Table S8. Collaborating centers at the RIS HPV TT and HPV VVAP study groups. [file CAM4-5-2909-s004.doc]

**Table S1: Sample distribution *per* anatomical location, geographical region and country**

| **Anatomical Location** | **Continent Category** | **Country** | **Number of Samples** |
| --- | --- | --- | --- |
| **Anal** | **Asia** | India | 1 |
|  |  | Korea | 20 |
|  | **Central/South America** | Chile | 2 |
|  |  | Colombia | 25 |
|  |  | Ecuador | 11 |
|  |  | Guatemala | 1 |
|  |  | Mexico | 26 |
|  |  | Paraguay | 7 |
|  | **Europe** | Czech Republic | 21 |
|  |  | France | 2 |
|  |  | Germany | 1 |
|  |  | Poland | 4 |
|  |  | Portugal | 20 |
|  |  | Slovenia | 12 |
|  |  | Spain | 19 |
|  |  | China | 1 |
| **Cervix** | **Asia** | India | 5 |
|  |  | Kuwait | 4 |
|  |  | Lebanon | 5 |
|  |  | Taiwan | 5 |
|  |  | Thailand | 1 |
|  |  | Turkey | 6 |
|  | **Central/South America** | Argentina | 15 |
|  |  | Brazil | 1 |
|  |  | Colombia | 15 |
|  |  | Guatemala | 11 |
|  |  | Mexico | 5 |
|  |  | Paraguay | 7 |
|  |  | Peru | 7 |
|  |  | Venezuela | 10 |
|  | **Europe** | France | 4 |
|  |  | Greece | 6 |
|  |  | Herzegovina | 6 |
|  |  | Italy | 13 |
|  |  | Netherlands | 7 |
|  |  | Poland | 17 |
|  |  | Portugal | 5 |
|  |  | Spain | 14 |
| **Penis** | **Asia** | India | 1 |
|  |  | Lebanon | 1 |
|  |  | Korea | 1 |
|  | **Central/South America** | Chile | 3 |
|  |  | Colombia | 12 |
|  |  | Ecuador | 9 |
|  |  | Guatemala | 2 |
|  |  | Honduras | 3 |
|  |  | Mexico | 4 |
|  |  | Paraguay | 7 |
|  |  | Venezuela | 2 |
|  | **Europe** | Czech Republic | 14 |
|  |  | Poland | 4 |
|  |  | Portugal | 3 |
|  |  | Spain | 36 |
|  |  | United Kingdom | 17 |
| **Vagina** | **Asia** | Bangladesh | 1 |
|  |  | India | 1 |
|  |  | Israel | 1 |
|  |  | Korea | 5 |
|  |  | Taiwan | 1 |
|  |  | Turkey | 1 |
|  | **Central/South America** | Argentina | 1 |
|  |  | Chile | 4 |
|  |  | Colombia | 8 |
|  |  | Ecuador | 14 |
|  |  | Guatemala | 2 |
|  |  | Uruguay | 1 |
|  |  | Mexico | 22 |
|  | **Europe** | Austria | 18 |
|  |  | Belarus | 3 |
|  |  | Czech Republic | 6 |
|  |  | France | 13 |
|  |  | Germany | 6 |
|  |  | Poland | 5 |
|  |  | Spain | 7 |
|  |  | United Kingdom | 2 |
| **Vulva** | **Asia** | India | 6 |
|  |  | Korea | 1 |
|  |  | Kuwait | 2 |
|  |  | Lebanon | 7 |
|  |  | Philippines | 1 |
|  |  | Taiwan | 2 |
|  |  | Turkey | 5 |
|  | **Central/South America** | Argentina | 2 |
|  |  | Brazil | 2 |
|  |  | Chile | 2 |
|  |  | Colombia | 8 |
|  |  | Ecuador | 5 |
|  |  | Guatemala | 1 |
|  |  | Honduras | 1 |
|  |  | Mexico | 5 |
|  |  | Paraguay | 5 |
|  |  | Uruguay | 3 |
|  |  | Venezuela | 2 |
|  | **Europe** | Austria | 14 |
|  |  | Belarus | 3 |
|  |  | Czech Republic | 5 |
|  |  | France | 3 |
|  |  | Germany | 10 |
|  |  | Greece | 2 |
|  |  | Italy | 5 |
|  |  | Poland | 4 |
|  |  | Portugal | 5 |
|  |  | Spain | 8 |
|  |  | United Kingdom | 9 |
|  |  | **Total** | **711** |

**Table S2: Primer design**

| **Target Gene** | **Amplified region**  **PCR product** |  | **Designed Primers** | **Specific SNPs** | **HPV16 (sub)variant** | |
| --- | --- | --- | --- | --- | --- | --- |
| **E6-1 ^a^** | nt 75-206 / 131 bp | F | 5’-GACATTTTMTGCACCAAAAGAGA-3’ | A83C G132C G132T T178G | | B  B  C  A4 |
|  |  | R | 5’-GCTTGCAGTACACACATTCTAATA-3’ |  |  |  |
| **E6-2 ^a^** | nt 274-388 / 114 bp | F | 5’-GAATCCATATGCTGTATGTGATAA-3’ | T350G | | A1 |
|  |  | R | 5’-CGGTTTGTTGTATTGCTGTT-3’ |  |  |  |
| **L2** | nt 5106-5312 / 206 bp | F | 5’-AGGCCAGCATTAACCTCTAGGCG-3’ | A5117G  T5138A  C5138A  C5144T  C5230G  A5258G  T5285A  A5286C  A5294C  T5309A | | C A1  A1  A1  D  C  D D D  D |
|  |  | R | 5’-AGTDGGTGAGGCTGCATGKGA-3’ |  |  |  |
| **LCR** | nt 7712-7876 / 164 bp | F | 5’- TGGCTTGTTTTAACTMMCCTAA -3’ | T7712A A7728C T7741G T7779C G7824A A7828C A7835C A7837G G7840A C7873G | | B A4 D A4 C A4 C C A4 A4 |
|  |  | R | 5’- KKTGTAACCCAAAATCGGT-3’ |  |  |  |

Table shows the target gene, the amplified region, the PCR product length, the designed primer sequence for each target gene and the lineage-specific polymorphism contained in each amplicon. F=Forward; R=Reverse. Reference sequence used for numbering: NC_001526. ^a^E6 primer sequence retrieved from Larsson and colleagues (22).

**Table S3: Likelihood weights for the attribution of each individual sequence to each (sub) variant, number of samples and percentage**

| **(sub)variant** | **Likelihood Ascription** | **Number of Samples** | **%** |
| --- | --- | --- | --- |
| **A1-3** | 0.6-0.7 | 11 | 1,84 |
|  | 0.70-0.89 | 99 | 16,56 |
|  | 0.90-0.94 | 129 | 21,57 |
|  | > 0.95 | 359 | 60,03 |
|  | Sub-total | 598 |  |
| **A4** | 0.6-0.7 | 1 | 3,23 |
|  | 0.70-0.89 | 2 | 6,45 |
|  | 0.90-0.94 | 0 | 0,00 |
|  | > 0.95 | 28 | 90,32 |
|  | Sub-total | 31 |  |
| **B** | 0.6-0.7 | 0 | 0,00 |
|  | 0.70-0.89 | 0 | 0,00 |
|  | 0.90-0.94 | 2 | 50,00 |
|  | > 0.95 | 2 | 50,00 |
|  | Sub-total | 4 |  |
| **C** | 0.6-0.7 | 0 | 0,00 |
|  | 0.70-0.89 | 0 | 0,00 |
|  | 0.90-0.94 | 0 | 0,00 |
|  | > 0.95 | 5 | 100,00 |
|  | Sub-total | 5 |  |
| **D** | 0.6-0.7 | 0 | 0,00 |
|  | 0.70-0.89 | 0 | 0,00 |
|  | 0.90-0.94 | 1 | 1,85 |
|  | > 0.95 | 53 | 98,15 |
|  | Sub-total | 54 |  |
| **Unclassified samples** | 0.6-0.7 | 1 | 5,26 |
|  | 0.70-0.89 | 8 | 42,11 |
|  | 0.90-0.94 | 6 | 31,58 |
|  | > 0.95 | 4 | 21,05 |
|  | Sub-total | 19 |  |
| **Total** |  | **711** |  |

**Table S4:** **Generalized Linear Model (GLM) and analysis of deviance**

|  | **Df** | **Res. Dev** | **Df** | **Res. Dev** | **p-value** | **% of total variance** |
| --- | --- | --- | --- | --- | --- | --- |
| **NULL** |  |  | 44 | 1289.29 |  |  |
| **Anatomical location** | 4 | 22.15 | 40 | 1267.14 | 0.0001867 | 1.72 |
| **Geography** | 2 | 182.06 | 38 | 1085.08 | < 2.2e-16 | 14.12 |
| **Variant** | 2 | 878.86 | 36 | 206.22 | < 2.2e-16 | 68.16 |
| **Anatomical location: Geography** | 8 | 37.75 | 28 | 168.47 | 8,38E-03 | 2.93 |
| **Anatomical location: Variant** | 8 | 35.57 | 20 | 132.90 | 2,10E-02 | 2.76 |
| **Geography: Variant** | 4 | 115.48 | 16 | 17.42 | < 2.2e-16 | 8.96 |
| **Anatomical location: Geography: Variant** | 16 | 17.42 | 0 | 0.00 | 0.3591931 | - |

Statistical mode approximation with the best fit for observed data for Europe, Central/ South America and Asia. Df= Degrees of freedom ; Res. Dev = Residual Deviance.

| **Continent** | **Variant** | **Cervix** | | **Vulva** | | **Vagina** | | **Penis** | | **Anus** | | **Total** | **χ2 test** |
| --- | --- | --- | --- | --- | --- | --- | --- | --- | --- | --- | --- | --- | --- |
|  |  | n | % | n | % | n | % | n | % | n | % | n |  |
| **Europe** | **A1-3** | 66 | 95,65 | 62 | 93,93 | 54 | 94,73 | 68 | 93,150 | 74 | 96,10 | 324 | 0,350 |
|  | **A4** | 0 | 0 | 2 | 3,03 | 0 | 0 | 0 | 0 | 2 | 2,53 | 4 |  |
|  | **D** | 3 | 4,35 | 2 | 3,03 | 3 | 5,26 | 5 | 6,84 | 1 | 1,29 | 14 |  |
| **Sub-total** |  | 69 |  | 66 |  | 57 |  | 73 |  | 77 |  |  |  |
| **Central/South**  **America** | **A1-3** | 54 | 78,26 | 28 | 87,50 | 43 | 89,58 | 33 | 82,50 | 67 | 93,05 | 225 | 0,110 |
|  | **A4** | 0 | 0 | 0 | 0 | 1 | 2,083 | 0 | 0 | 0 | 0 | 1 |  |
|  | **D** | 15 | 21,73 | 4 | 12,50 | 4 | 8,33 | 7 | 17,50 | 5 | 6,94 | 35 |  |
| **Sub-total** |  | 69 |  | 32 |  | 48 |  | 40 |  | 72 |  |  |  |
| **Asia** | **A1-3** | 20 | 80 | 17 | 73,91 | 6 | 66,66 | 2 | 100 | 4 | 19,04 | 49 | 0,002/ < 0.0005† |
|  | **A4** | 3 | 12 | 3 | 13,04 | 3 | 33,33 | 0 | 0 | 17 | 80,95 | 26 |  |
|  | **D** | 2 | 7,69 | 3 | 13,04 | 0 | 0 | 0 | 0 | 0 | 0 | 5 |  |
| **Sub-total** |  | 25 |  | 23 |  | 9 |  | 2 |  | 21 |  |  |  |
| **Total** |  | 163 |  | 121 |  | 114 |  | 115 |  | 170 |  | 683 |  |
| **Fisher test** |  | <0,0005/0,003 | | 0,02/ 0,182 | | 0,004 /0,562 | | 0,340/0,114 | | <0,0005/0,059 | |  |  |

**Table S5: HPV16 A1-3, A4 and D variant distribution by anatomical location within each geographical area**

The contingency table shows HPV16 variants distribution for the 683 samples analysed, according to geographical region and anatomical location. Differences in variant prevalence between anatomical sites within a given geographical region are given for each row (χ2 test ). Differences in variant prevalence between geographical regions, within a given anatomical location are given for each column (Fisher test). Abbreviations: A1-3= HPV16_A1, HPV16_A2 and HPV16_A3; A4= HPV16_A4; D=HPV16_D †p-value for the χ2 test b excluding vaginal and penile samples from Asia, both present in low numbers.

**Table S6 : Age at tumour diagnosis for invasive squamous invasive carcinomas HPV positive, HPV negative, HPV16 single infected and HPV16 A1-A2-A3 stratified by cervix, non-cervix (women) and anogenital (men) samples**

| **Age of tumour diagnosis** | | | | | | | |
| --- | --- | --- | --- | --- | --- | --- | --- |
| **SCC** | **Anatomical location** | **Median ± MAD** | **Quartile 1** | **Quartile 3** | **Range**† | **number** | **Wilcox. p-value** |
| **HPV positive** | **cervix** | 50±13 | 42 | 60 | (17-87) | 6888 | Ref |
|  | **non-cervix (women)** | 64±17 | 51 | 75 | (30-106) | 968 | p<0.0005 |
|  | **anogenital (men)** | 62±18 | 50 | 73 | (24-97) | 376 | p<0.0005 |
| **HPV16 single infected** | **cervix** | 49±13 | 40 | 59 | (17-87.5) | 4032 | Ref |
|  | **non-cervix (women)** | 65±16 | 53 | 75 | (22-106) | 656 | p<0.0005 |
|  | **anogenital (men)** | 63±16 | 52 | 74 | (25-97) | 245 | p<0.0005 |
| **HPV16 A1-3** | **cervix** | 48±13 | 41 | 60 | (25-85) | 107 | Ref |
|  | **non-cervix (women)** | 63±16 | 52 | 74 | (25-97) | 304 | p<0.0005 |
|  | **anogenital (men)** | 66.5±14 | 55 | 74 | (35-97) | 134 | p<0.0005 |

Table represent the median , the 25% and 75% quatiles, range and number of samples. Median differences between cervix (Reference) and the other anogenital locations are performed by Wilcoxon Mann-Whitney test. HPV positive samples include all the types detectable though SPF_10_-LiPA_25_ protocol (version 1; Laboratory Biomedical Products, Rijswijk, Netherlands) † Range, 1.5x Inter-quartile

**Table S7 : Age at tumour diagnosis for invasive squamous invasive carcinomas HPV positive, HPV16 single infected and HPV16 A1-A2-A3 stratified by anatomical location**

| **Age of tumour diagnosis** | | | | | | | | |
| --- | --- | --- | --- | --- | --- | --- | --- | --- |
| **SCC** | **Antomical location** | **Median ± MAD** | **Quartile 1** | **Quartile 3** | | **Range**† | **number** | **Wilcox. p-value** |
| **HPV positive** | **Cervix** | 50±13 | 42.00 | | 60.00 | (17-87) | 6888 | Ref |
|  | **Vulva** | 64±19 | 50.00 | | 76.00 | (20-97) | 444 | p<0.0005 |
|  | **Vagina** | 62±16 | 51.5 | | 72.5 | (22-94) | 275 | p<0.0005 |
|  | **Penis** | 65±18 | 52.00 | | 75.00 | (24-97) | 276 | p<0.0005 |
|  | **Anus (men)** | 58.5±14 | 45.75 | | 68 | (31-89) | 100 | p<0.0005 |
|  | **Anus (women)** | 66±15 | 54 | | 75 | (30-106) | 249 | p<0.0005 |
| **HPV16 single infected** | **Cervix** | 49±13 | 40.00 | | 59.00 | (17-87.5) | 4033 | Ref |
|  | **Vulva** | 62±19 | 50.00 | | 76.00 | (22-97) | 294 | p<0.0005 |
|  | **Vagina** | 62.5±16 | 52.25 | | 72.75 | (28-94) | 162 | p<0.0005 |
|  | **Penis** | 65±16 | 53.00 | | 75.00 | (25-97) | 175 | p<0.0005 |
|  | **Anus (men)** | 61.5±16 | 49.00 | | 68.75 | (35-89) | 70 | p<0.0005 |
|  | **Anus (women)** | 67±14 | 56.00 | | 75.00 | (30-90) | 200 | p<0.0005 |
| **HPV16 A1-3** | **Cervix** | 49±13 | 41.00 | | 60.00 | (25-85) | 107 | Ref |
|  | **Vulva** | 62±21 | 50.75 | | 80.00 | (25-97) | 100 | p<0.0005 |
|  | **Vagina** | 64±15 | 54.00 | | 74.00 | (32-92) | 102 | p<0.0005 |
|  | **Penis** | 68.5±16 | 56.75 | | 76.25 | (36-97) | 96 | p<0.0005 |
|  | **Anus (men)** | 63.5±13 | 49.00 | | 68.75 | (35-81) | 38 | p<0.0005 |
|  | **Anus (women)** | 65±13 | 54.00 | | 74.00 | (35-90) | 102 | p<0.0005 |

Table represent the median , the 25% and 75% quatiles, range and number of samples. Median differences between cervix (Reference) and the other anogenital locations are performed by Wilcoxon Mann-Whitney test. HPV positive samples include all the types detectable though SPF_10_-LiPA_25_ protocol (version 1; Laboratory Biomedical Products, Rijswijk, Netherlands). †Range, 1.5xInter-quartile

| **Table S8**: **Collaborating centres at the RIS HPV TT and HPV VVAP study groups** |
| --- |
| **1.RIS HPV TT study group:** |
| 1. Algeria: Doudja Hammouda (National Institute of Health, Registre des Tumeurs d’Alger), Anissa Bouhadef (C.H.U Hussein-Dey- Hospital Nafissa Hamoud). |
|  |
| 2. Argentina: Silvio Alejandro Tatti, Susana Vighi (Hospital de Clínicas José de San Martín, Universidad de Buenos Aires). |
|  |
| 3. Australia: Suzanne M. Garland, Sepher Tabrizi (The Royal Women’s Hospital, The University of Melbourne). |
|  |
| 4. Bangladesh: Ashrafun Nessa, AJE Nahar Rahman, Faruk Ahmed, Mohammad Kamal (Bangabandhu Sheikh Mujib Medical University – BSMMU). |
|  |
| 5. Bosnia Herzegovina: Ermina Iljazovic (University Clinical Center Tuzla BiH). |
|  |
| 6. Brazil: Marcus Aurelho de Lima, Sérgio Henrique (Laboratório de Anatomia |
| Patológica e Citologia Ltda, Associação de Combate ao Câncer do Brasil Central, Hospital Dr. Hélio Angotti, PATMED). |
|  |
| 7. Chile: Rodrigo Prado (Centro de Oncología Preventiva, Facultad de Medicina, Universidad de Chile), Ximena Rodriguez, Marisol Guerrero,Virginia Leiva, Elsa Olave, Claudia Ramis, Viviana Toro (Complejo Hospitalario San José). |
|  |
| 8. China: You Lin Qiao, Chen Wen, Wu Su Hui (Cancer Institute, Chinese Academy of Medical Sciences and Peking Union Medical College). |
|  |
| 9. Colombia: Hector Jaime Posso (Liga Contra el Cáncer de Bogotá), Luis Eduardo Bravo, Tito Collazos, Luz Stella García (Hospital Universitario del Valle), Raúl Murillo, Gustavo Adolfo Hernández Suárez, Carlos Eduardo Pinzón (Instituto Nacional de Cancerología), Gloria I. Sánchez (Universidad de Antioquía). |
|  |
| 10. Croatia: Magdalena Grce, Ivan Sabol (Rudjer Boskovic Institutem), Sonja Dzebro, Mara Dominis (Clinical Hospital Merkur). |
|  |
| 11. Czech Republic: Ivo Steiner (Faculty of Medicine and Faculty Hospital, Hradec Kralove), Vladimir Vonka (Institute of Hematology and Blood Transfusion). |
|  |
| 12. France: Christine Clavel (CHU Reims, Laboratoire Pol Bouin, Hôpital Maison Blanche), Massimo Tommasino (International Agency for Research on Cancer). |
|  |
| 13. Greece: Maria Tzardi (Medical School of University of Crete), Theodoros Agorastos (Aristotle University of Thessaloniki). |
|  |
| 14. Guatemala: Luis Estuardo Lombardi, Edgar Kestler, Obdulia Salic, Sergio Marroquin, Victor Argueta (Centro de Investigación Epidemiológica en Salud Sexual y Reproductiva - CIESAR, Hospital General San Juan de Dios), Walter Guerra (Instituto Nacional del Cáncer), Hesler Morales (Instituto Guatemalteco de Seguridad Social, Instituto Nacional del Cáncer). |
|  |
| 15. Honduras: Annabelle Ferrera (Escuela de Microbiología, Universidad Nacional Autónoma de Honduras), Ricardo Bulnes (Hospital General San Felipe). |
|  |
| 16. India: Asha Jain (Cancer Prevention and Relief Society Raipur), PSA Sarma (BSP Hospital), Sushil K Giri (Regional Cancer Center); Maheep Bhalla (JLN Hospital & Research Center), Bharat Patel (Lab One). |
|  |
| 17. Italy: Luciano Mariani, Ferdinando Marandino (Regina Elena Cancer Institute). |
|  |
| 18. Israel: Jacob Bornstein, Alejandro Livoff, Hector Itzhac Cohen (Western Galilee Hospital). |
|  |
| 19. Japan: Toshiyuki Sasagawa (Kanazawa University Hospital), Shintaro Terahata (Tonami General Hospital), Kazuhisa Ishi (Juntendo University, Urayasu Hospital). |
|  |
| 20. South Korea: Hai-Rim Shin, Jin-Kyoung Oh (National Cancer Center), Jung-II Suh (National Medical Center), and Seo-Hee Rha (Dong-A University). |
|  |
| 21. Kuwait: Waleed Fahad Al-Jassar (Faculty of Medicine, Kuwait University), Rema’a Al-Safi (Maternity Hospital). |
|  |
| 22. Lebanon: Muhieddine Seoud (The American University of Beirut Medical Center). |
|  |
| 23. México: Patricia Alonso de Ruiz, Gustavo Lastra, Alma Karina Olivares Montano (Hospital General de México, Facultad de Medicina, Universidad Nacional Autónoma de México). |
|  |
| 24. Mozambique: Clara Menéndez, Cleofé Romagosa, Carla Carrillo (Barcelona Center for International Health Research, Hospital Clínic/Universitat de Barcelona, and Manhiça Health Research Center). |
|  |
| 25. Nigeria: Adekunbiola Banjo, Rose Anorlu, Fatimah B. Abdulkareem, A.O Daramola, CC Anunobi (Lagos University Teaching Hospital Idi-Araba). |
|  |
| 26. Paraguay: Elena Kasamatsu, Francisco Perrota (Instituto de Investigaciones en Ciencias de la Salud, Universidad Nacional de Asunción). |
|  |
| 27. Perú: Carlos Santos, Eduardo Cáceres, Henry Gómez, Juvenal Sánchez, Carlos S. Vallejos, (Instituto Nacional de Enfermedades Neoplásicas). |
|  |
| 28. The Philippines: Efren J. Domingo, María Julieta V. Germar, Jeric**h**o Thaddeus P. Luna, Carolyn Zalameda-Castro, Arnold M. Fernandez, Roslyn Balacuit (University of the Philippines College of Medicine); Cecilia Ladines Llave, Jean Anne Toral (Cervical Cancer Prevention Center – CECAP, Cancer Institute (UP-CM-PGH)). |
| 29. Poland: Andrzej Marcin Nowakowski (Medical University of Lublin). |
|  |
| 30. Portugal: Eugenia Cruz (Centro Regional de Oncologia Coimbra, Instituto Português de Oncologia); Manuela Lacerda, Manuel Sobrinho-Simoes (Institute of Molecular Pathology and Immunology of the University of Porto); Ana Felix (Instituto Portugues de Oncologia de Lisboa Francisco Gentil). |
|  |
| 31. Spain: Enrique Lerma (Hospital de la Santa Creu i Sant Pau); Enrique Poblet (Hospital General Universitário de Albacete); Lluís Eleuteri Pons (Hospital de Tortosa Verge de La Cinta); Antonio Llombart-Bosch, Morelva Toro de Méndez (Facultad de Medicina, Universidad de Valencia); Belen Lloveras (Hospital del Mar); Ana Puras Gil (Hospital Virgen del Camino); Miguel Andújar (Complejo Hospitalario Universitario Insular Marteno-Infantil); Jaume Ordi (CRESIB - Hospital Clínic); Adela Pelayo (Clinica San Carlos); Julio Velasco, Cristina Pérez (Hospital San Agustín, & IUOPA (Oncologic and Universitary Institute of Principality of Astúrias)); Maria Alejo (Hospital General de l’Hospitalet); Ignacio G. Bravo (CSISP - Centre for Public Health Research, Centro Superior de Investigación en Salud Pública, Conselleria de Sanidad (Generalitat Valenciana)); Laia Alemany, F. Xavier Bosch, Vanesa Camón, Gabriel Capellà, Cristina Caupena, Xavier Castellsagué, Omar Clavero, Silvia de Sanjosé, Mireia Diaz, Ana Esteban, Rebeca Font, Jose M. Godínez, Nuria Guimerà, Yolanda Florencia, Helena Frayle, Mercedes Hurtado, Joellen Klaustermeier, Anna Merchán, Carles Miralles, Nuria Monfulleda, Nubia Muñoz, Bea Quirós Cristina Rajo, Sara Tous, Marleny Vergara (IDIBELL, Institut Català d’Oncologia (ICO) - Catalan Institute of Oncology); August Vidal (Hospital Universitari de Bellvitge). |
|  |
| 32. Taiwan: Chou Cheng-Yang (National Cheng Kung University Medical College, Taiwan Association of Gynecologic Oncologists); Tang-Yuan Chu (Buddhist Tzuchi Genral Hospital); Kuo-Feng Huang (Chi Mei Medical Center); Cheng Wen-Fang (National Taiwan University Hospital); Chih- Ming HO (Gynecologic Cancer Center, Cathay General Hospital). |
|  |
| 33. Thailand: Saibua C. Bunnag Chichareon, Kobkul Tungsinmunkong, Jintamard Suwanjarat (Prince of Songkla University). |
|  |
| 34. The Netherlands: Chris J.L.M Meijer, Peter J.F Snijders (Vrije Universiteit Medical Center); Wim G.V. Quint, Jean-Paul Brunsveld, Anco C. Molijn, Daan T. Geraets (DDL Diagnostic Laboratory). |
|  |
| 35. Turkey: Alp Usubutun (Medical School, Hacettepe University). |
|  |
| 36. Uganda: Michael Odida (Uganda Makerere University); Elisabete Weiderpass (Karolinska Institutet, Sweden; The Norwegian Cancer Registry, Norway; Samfunded Folkhalsan, Finland). |
|  |
| 37. United States of America: Esther Oliva (Massachusetts General Hospital); Thomas C. Wright (New York Presbyterian Hospital, Columbia University Medical Center). |
|  |
| 38. Venezuela: Enrique López Loyo (Sociedad Venezolana de Patología); Victoria García Barriola, Mirian Naranjo de Gómez, Adayza Figueredo, Janira Navarro (Universidad Central de Venezuela). |
|  |
| **2.HPV VVAP study group for vulvar site, updated September 2012:** |
| 1.      Argentina: Myriam Perrotta, Ana Jaen, Kevin Davies, Henry Kitchener, Godfrey Wilson (Hospital Italiano de Buenos Aires); |
|  |
| 2.      Australia: Suzanne M. Garland, Sepher N. Tabrizi (The Royal Women’s Hospital, The University of Melbourne); Gerard Vincent Wain, Catherine Jane Kennedy, Yoke-Eng Chiew (Gynaecological Oncology, Westmead Hospital); Raghwa Sharma (Department of Tissue Pathology and Diagnostic Oncology, University of Sydney and University of Western Sydney Westmead Hospital); |
|  |
| 3.      Austria: Elmar Armin Joura (University Hospital and Medical School); |
|  |
| 4.      Bangladesh: Ashrafun Nessa, AJE Nahar Rahman, Mohammed Kamal (Bangabandhu Sheikh Mujib Medical University – BSMMU); Faruk Ahmed (Dhaka Medical College Hospital); |
|  |
| 5.      Belarus: Halina Viarheichyk, Sitnikov Valeriy (Gomel State Medical University); Achynovich Searhei (Gomel Regional Clinical Oncological Hospital); |
|  |
| 6.      Bosnia Herzegovina: Ermina Iljazovic ( University Clinical Center Tuzla BiH); |
|  |
| 7.      Brazil: Paula Maldonado, Gutemberg Leão Almeida, Isabel Val, Renata Fonseca, Roberto José Lima, Marcia Mannarino, Yara Furtado (Instituto de Ginecologia da Universidad Federal do Rio de Janeiro); |
|  |
| 8.      Chile: Rodrigo Prado, Carla Molina, rosa Muñoz (Centro de Oncología Preventiva, Facultad de Medicina, Universidad de Chile); Ximena Rodriguez, Marisol Guerrero,Virginia Leiva, Elsa Olave, Claudia Ramis, Viviana Toro (Hospital de San José); |
|  |
| 9.      Colombia: Raúl Murillo, Gustavo Adolfo Hernández Suárez, Carlos Eduardo Pinzón (Instituto Nacional de Cancerología); Czech Republic: Václav Mandys (3rd Faculty of Medicine and University Hospital King’s Wineyards); Jan Laco (Faculty Hospital Hradec Kralove); |
|  |
| 10.  Ecuador: Leopoldo Tinoco, Hospital Oncológico de Quito, Quito, Ecuador; |
|  |
| 11.  France: Christine Clavel, Philippe Birembaut, Veronique Dalstein (CHU de Reims, Laboratoire Pol Bouin/ INSERM UMR-S 903, Reims); Christine Bergeron (Laboratoire Cerba, Department de Pathology, Cergy Pontoise); Massimo Tommasino (International Agency for Research on Cancer); |
|  |
| 12.  Germany: Monika Hampl, Pof. Baldus (University Hospital of Duesseldorf); Karl Ulrich Petry, Alexander Luyten (Klinikum Wolfsburg); Michael Pawlita, Gordana Halec (Department Genome Changes and Carcinogenesis. Heildelberg); |
|  |
| 13.  Greece: Theodoros Agorastos (Aristotle University of Thessaloniki); |
|  |
| 14.  Guatemala: Luis Estuardo Lombardi, Edgar Kestler, Obdulia Salic, Sergio Marroquin, Victor Argueta (Centro de Investigación Epidemiológica en Salud Sexual y Reproductiva-CIESAR, Hospital General San Juan de Dios); Walter Guerra (Instituto Nacional del Cáncer); Hesler Morales (Instituto Guatemalteco de Seguridad Social, Instituto Nacional del Cáncer); |
|  |
| 15.  Honduras: Annabelle Ferrera (Escuela de Microbiología, Universidad Nacional Autónoma de Honduras); |
|  |
| 16.  India: Neerja Bhatla (Institute of Medical Science New Dehli); |
|  |
| 17.  Israel: Jacob Bornstein, Alejandro Livoff, Hector Itzhac Cohen (Western Galilee Hospital- Nahariya); |
|  |
| 18.  Italy: Luciano Mariani, Amina Vocaturo, Maria Benevolo, Fernando Marandino, Francesca Rollo (Regina Elena Cancer Istitute); |
|  |
| 19.  Korea-South: Hai-Rim Shin, Jin-Kyung Oh (National Cancer Center); Shin Gwang Kang (Asian Medical Center); Dong-chul Kim (Kangnam St. Mary's Hospital); |
|  |
| 20.  Kuwait: Waleed Al-Jassar (Faculty of Medicine, Kuwait University), Rema'a Al-Safi (Maternity Hospital); |
|  |
| 21.  Lebanon: Muhieddine Seoud (The American University of Beirut Medical Center); Mali: Bakarou Kamate, Cathy Ndiaye (Hospital National DU Point G); |
|  |
| 22.  Mexico: Isabel Alvarado-Cabrero (Instituto Mexicano del Seguro Social); Rubén López-Revilla, Claudia Magaña-León (Instituto Potosino de Investigación Científica y Tecnológica, AC); Cuauhtémoc Oros (Hospital Central Ignacio Morones Prieto, San Luis Potosí); |
|  |
| 23.  Mozambique: Carla Carrilho (Eduardo Mondlane University); |
|  |
| 24.  New Zealand: Susan M Bigby, RW Jones, KL Fong, D Rowan, J Baranyai, L Eva (Middlemore Hospital); |
|  |
| 25.  Nigeria: A.A.F. Banjo, F.B. Abdulkareem, A.O. Daramola, C.C. Anunobi, R.U. Anorlu (Lagos University Teaching Hospital Idi-Araba); Sani Malami, Ali Bala Umar (Faculty of Medicine, Bayero University); |
|  |
| 26.  Paraguay: Elena Kasamatsu, Antonio Leoploldo Cubilla, Francisco Perrota (Instituto de Investigaciones en Ciencias de la Salud, Universidad Nacional de Asunción); |
|  |
| 27.  Philippines: Celia Ladines Llave, Jean Anne Toral (Cervical Cancer Prevention Center-CECAP, Cancer Institute (UP-CM-PGH)); Efren j Domingo, Maria Julieta V. Germar, Jerico Thaddeus, P. Luna, Arnold M. Fernandez, Carolyn Zalameda Castro, Roslyn Balacuit (University of the Philippine College of Medicine General Hospital); |
|  |
| 28.  Poland: Andrzej Marcin Nowakowski (Medical University of Lublin); Robert Jach, Jolanta Orlowska-Heitzman, Monika Kabzinska-Turek, Paulina Przybylska, Marzena kula-Prykan (Jagiellonian University Medical College); |
|  |
| 29.  Portugal: Eugenia Cruz (Centro Regional de Oncologia Coimbra, Instituto Português de Oncologia); Ana Félix, Jorge Manuel Soares (Instituto Portugues de Oncologia de Lisboa Francisco Gentil); |
|  |
| 30.  Senegal: Cathy Ndiaye, Nafissatou Ndiaye Ba, Victorino Mendes (HOGGY stands for Hôpital Général de Grand Yoff; DANTEC - Hôpital A. Le Dantec; FAC - Faculté de Médecine - Université Cheikh A. Diop); |
|  |
| 31.  Spain: Maria Alejo (Hospital General d’Hospitalet); Belén Lloveras (Hospital del Mar); Laia Alemany, F. Xavier Bosch, Ignacio Bravo, Vanesa Camón,  Xavier Castellsagué, Omar Clavero, Silvia de Sanjosé,  Ana Esteban, Jose M. Godínez, Yolanda Florencia, Joellen Klaustermeier, Nubia Muñoz, Beatriz Quirós, Maëlle Saunier, Cristina Rajo, Sara Tous, Marleny Vergara (IDIBELL, Institut Català d’Oncologia – Catalan Institute of Oncology), August Vidal, Enric Condom (Hospital Universitari de Bellvitge), Jaume Ordi (Hospital Clínic), Julio Velasco, Cristina Pérez (Hospital San Agustín); |
|  |
| 32.  Taiwan: Chou Cheng-Yang (National Cheng Kung University Medical College,Taiwan Association of Gynecologic Oncologists); Tang-Yuan Chu (Buddhist Tzuchi Genral Hospital); Kuo-Feng Huang (Chi Mei Medical Center); Cheng Wen-Fang (National Taiwan University Hospital); Chih- Ming HO (Gynecologic Cancer Center, Cathay General Hospital); |
|  |
| 33.  The Netherlands: Wim Quint, , Anco C. Molijn, Daan T. Geraets, Nuria Guimera (DDL Diagnostic Laboratory); (Chris J.L.M Meijer (Vrije Universiteit Medical Center); |
|  |
| 34.  Turkey: Alp Usubutun (Hacettepe University); UK: Henry Kitchener (School of Medicine, University of Manchester); Robyn Davies (Manchester Royal Infirmary); Paul Cross (Queen Elizabeth Hospital, Sheriff Hill); |
|  |
| 35.  Uruguay: Adela Rosa Sica, Benedicta Caserta, Mabel Cedeira, Daniel Mazal, Guillermo Rodríguez (Laboratorio de Anatomía patológica del hospital de la Mujer, Montevideo); |
|  |
| 36.  USA: Wendy Cozen, Marc T. Goodman, Brenda Y. Hernández, Charles Lynch, Daniel B. Olson, Freda R. Selk (Cancer Center, Hawaii-Iowa); |
|  |
| 37.  Venezuela: Enrique López Loyo (Sociedad Venezolana de Patología); Victoria García Barriola, Mirian Naranjo de Gómez, Adayza Figueredo, Janira Navarro (Universidad Central de Venezuela). |
|  |
| **3.HPV VVAP study group for vagina site, updated December 2013:** |
| 1.      Argentina: Myriam Perrotta, Ana Jaen, Kevin Davies (Hospital Italiano de Buenos Aires); |
|  |
| 2.      Australia: Suzanne M. Garland, Sepehr N. Tabrizi (The Royal Women’s Hospital, The University of Melbourne); Gerard Vincent Wain, Catherine Jane Kennedy, Yoke-Eng Chiew (Gynaecological Oncology, Westmead Hospital); Raghwa Sharma (Department of Tissue Pathology and Diagnostic Oncology, University of Sydney and University of Western Sydney Westmead Hospital); |
|  |
| 3.      Austria: Elmar Armin Joura (University Hospital and Medical School); Josefine Stani and Reinhard Horvat, MD (Medical University and General Hospital Vienna, Austria); |
|  |
| 4.      Bangladesh: Ashrafun Nessa, AJE Nahar Rahman, Mohammed Kamal (Bangabandhu Sheikh Mujib Medical University – BSMMU); Faruk Ahmed (Dhaka Medical College Hospital); |
|  |
| 5.      Belarus: Halina Viarheichyk, Sitnikov Valeriy (Gomel State Medical University); Achynovich Searhei (Gomel Regional Clinical Oncological Hospital); |
|  |
| 6.      Brazil: Paula Maldonado, Gutemberg Leão Almeida, Isabel Val, Renata Fonseca, Roberto José Lima, Marcia Mannarino, Yara Furtado (Instituto de Ginecologia da Universidad Federal do Rio de Janeiro); |
|  |
| 7.      Chile: Rodrigo Prado, Carla Molina, Rosa Muñoz (Centro de Oncología Preventiva, Facultad de Medicina, Universidad de Chile); Ximena Rodriguez, Marisol Guerrero,Virginia Leiva, Elsa Olave, Claudia Ramis, Viviana Toro (Hospital de San José); |
|  |
| 8.      Colombia: Raúl Murillo, Gustavo Adolfo Hernández Suárez, Carlos Eduardo Pinzón, Nubia Muñoz (Instituto Nacional de Cancerología); |
|  |
| 9.      Czech Republic: Václav Mandys (3rd Faculty of Medicine and University Hospital King’s Wineyards); Jan Laco (The Fingerland Department of Pathology, Charles University in Prague, Faculty of Medicine and University Hospital Hradec Kralove); |
|  |
| 10.  Ecuador: Leopoldo Tinoco (Hospital Oncológico de Quito, Quito, Ecuador); |
|  |
| 11.  France: Christine Clavel, Philippe Birembaut, Véronique Dalstein (CHU de Reims, Laboratoire Pol Bouin / INSERM UMR-S 903, Reims, France); Christine Bergeron (Laboratoire Cerba, Department de Pathology, Cergy Pontoise); Massimo Tommasino (International Agency for Research on Cancer); |
|  |
| 12.  Germany: Karl Ulrich Petry, Alexander Luyten (Klinikum Wolfsburg); Michael Pawlita, Gordana Halec, Dana Holzinger (Department Genome Changes and Carcinogenesis, Heildelberg); |
|  |
| 13.  Greece: Theodoros Agorastos (Aristotle University of Thessaloniki); |
|  |
| 14.  Guatemala: Luis Estuardo Lombardi, Edgar Kestler, Obdulia Salic, Sergio Marroquin, Victor Argueta (Centro de Investigación Epidemiológica en Salud Sexual y Reproductiva-CIESAR, Hospital General San Juan de Dios); Walter Guerra (Instituto Nacional del Cáncer); Hesler Morales (Instituto Guatemalteco de Seguridad Social, Instituto Nacional del Cáncer; Instituto de Cancerologia Dr. Bernardo del Valle S); |
|  |
| 15.  India: Asha Jain (Cancer Prevention and Relief Society Raipur); Sushil K Giri (Regional Cancer Center, Cuttack); Maheep Bhalla (JLN Hospital & Research Center,BSP, Bhilai); Bharat Patel (Lab One Raipur); PSA Sarma (BSP Hospital); |
|  |
| 16.  Israel: Jacob Bornstein, Alejandro Livoff, Hector Itzhac Cohen (Western Galilee Hospital- Nahariya); |
|  |
| 17.  Korea-South: Hai-Rim Shin, Jin-Kyung Oh (National Cancer Center); Shin Gwang Kang (Asian Medical Center); Dong-chul Kim (Kangnam St. Mary's Hospital); |
|  |
| 18.  Kuwait: Dr. Waleed Al- Jassar.  Faculty of Medicine, Kuwait University, Dr. Rema'a Al-Safi. Maternity Hospital, Kuwait; |
|  |
| 19.  Mexico: Isabel Alvarado-Cabrero (Instituto Mexicano del Seguro Social); Rubén López-Revilla, Claudia Magaña-León (Instituto Potosino de Investigación Científica y Tecnológica, AC); Cuauhtémoc Oros (Hospital Central Ignacio Morones Prieto, San Luis Potosí); |
|  |
| 20.  Mozambique: Carla Carrilho (Eduardo Mondlane University); |
|  |
| 21.  Nigeria: Adekunbiola A.F. Banjo, F.B. Abdulkareem, A.O. Daramola, C.C. Anunobi, R.U. Anorlu (Lagos University Teaching Hospital Idi-Araba); |
|  |
| 22.  Paraguay: Elena Kasamatsu, Antonio Leoploldo Cubilla, Francisco Perrota (Instituto de Investigaciones en Ciencias de la Salud, Universidad Nacional de Asunción, Instituto de Patología e Investigación); |
|  |
| 23.  Philippines: Celia Ladines Llave, Jean Anne Toral (Cervical Cancer Prevention Center, Cancer Institute); Efren Javier Domingo, Jericho Thaddeus P. Luna, Maria Julieta V. Germar, Arnold M. Fernandez, Carolyn Zalameda Castro, Roslyn Balacuit (University of the Philippine College of Medicine General Hospital); |
|  |
| 24.  Poland: Andrzej Marcin Nowakowski (Medical University of Lublin); Robert Jach, Jolanta Orlowska-Heitzman, Monika Kabzinska-Turek, Paulina Przybylska, Marzena kula-Prykan (Jagiellonian University Medical College); |
|  |
| 25.  Spain: Belén Lloveras (Hospital del Mar); August Vidal, Enric Condom (Hospital Universitari de Bellvitge); Jaume Ordi (Hospital Clínic); Julio Velasco Alonso, Cristina Pérez (Hospital San Agustín); Maria Alejo (Hospital General de l’Hospitalet, Barcelona); Laia Alemany, Francesc Xavier Bosch, Ignacio G. Bravo, Vanesa Camón, Xavier Castellsagué, Omar Clavero, Silvia de Sanjosé, Ion Espuña, Anna Esteban, José M. Godínez, Yolanda Florencia, Klaustermeier, Natividad Patón, Beatriz Quirós, Cristina Rajo, Maëlle Saunier, Sara Tous, Marleny Vergara (IDIBELL, Institut Català d’Oncologia-Catalan Institute of Oncology); |
|  |
| 26.  Taiwan: Chou Cheng-Yang (National Cheng Kung University Medical College,Taiwan Association of Gynecologic Oncologists); Tang-Yuan Chu (Buddhist Tzuchi Genral Hospital); Kuo-Feng Huang (Chi Mei Medical Center); Cheng Wen-Fang (National Taiwan University Hospital); Chih-Ming HO (Gynecologic Cancer Center, Cathay General Hospital); |
|  |
| 27.  The Netherlands: Wim G.V. Quint, Anco C. Molijn, Daan T. Geraets, Núria Guimerà (DDL Diagnostic Laboratory); Chris J.L.M Meijer (Vrije Universiteit Medical Center); |
|  |
| 28.  Turkey: Alp Usubutun (Hacettepe University); |
|  |
| 29.  UK: Henry Kitchener, Godfrey Wilson (School of Medicine, University of Manchester); Paul Cross (Queen Elizabeth Hospital, Sheriff Hill); |
|  |
| 30.  Uruguay: Adela Rosa Sica, Benedicta Caserta, Mabel Cedeira, Daniel Mazal, Guillermo Rodríguez (Laboratorio de Anatomía Patológica del Hospital de la Mujer, Montevideo); |
|  |
| 31.  USA: Marc T. Goodman, Wendy Cozen (Cedars Sinai Medical Center, Los Angeles, California); Marc T. Goodman, Brenda Y. Hernández (Cancer Center, Hawaii); Charles F. Lynch, Daniel B. Olson, Freda R. Selk (Iowa); |
|  |
| 32.  Venezuela: Enrique López Loyo (Sociedad Venezolana de Patología); Victoria García Barriola, Mirian Naranjo de Gómez, Adayza Figueredo, Janira Navarro (Universidad Central de Venezuela). |
|  |
| 33.  The advisory committee members are: Chris J Meijer, Massimo Tommasino, Michael Pawlita, Wim Quint and Nubia Muñoz |
|  |
| 4.**HPV VVAP study group for penile site, updated November 2014:** |
|  |
| 1.      Australia: Gerard Vincent Wain, Catherine Jane Kennedy, Yoke-Eng Chiew (Gynaecological Oncology, Westmead Hospital); Raghwa Sharma (Department of Tissue Pathology and Diagnostic Oncology, University of Sydney and University of Western Sydney Westmead Hospital); |
|  |
| 2.      Bangladesh: Ashrafun Nessa, AJE Nahar Rahman, Mohammed Kamal (Bangabandhu Sheikh Mujib Medical University – BSMMU); Faruk Ahmed (Dhaka Medical College Hospital); |
|  |
| 3.      Chile: Rodrigo Prado, Carla Molina, rosa Muñoz (Centro de Oncología Preventiva, Facultad de Medicina, Universidad de Chile); Ximena Rodriguez, Marisol Guerrero,Virginia Leiva, Elsa Olave, Claudia Ramis, Viviana Toro (Hospital de San José); |
|  |
| 4.      Colombia: Raúl Murillo, Gustavo Adolfo Hernández Suárez, Carlos Eduardo Pinzón (Instituto Nacional de Cancerología); |
|  |
| 5.      Czech Republic: Václav Mandys (3rd Faculty of Medicine and University Hospital King’s Wineyards); Jan Laco (Faculty Hospital Hradec Kralove); |
|  |
| 6.      Ecuador: Leopoldo Tinoco (Hospital Oncológico Solca-Quito); |
|  |
| 7.      France: Christine Clavel, Philippe Birembaut, Veronique Dalstein (CHU de Reims, Laboratoire Pol Bouin/ INSERM UMR-S 903, REIMS); Christine Bergeron (Laboratoire Cerba, Department de Pathology, Cergy Pontoise); Massimo Tommasino (International Agency for Research on Cancer); |
|  |
| 8.      Germany: Michael Pawlita, Gordana Halec (Department Genome Changes and Carcinogenesis. Im Neuenheimer Feld 242. D-69120 Heildelberg); |
|  |
| 9.      Greece: Maria Tzardi (Medical School of University of Crete); |
|  |
| 10.  Guatemala: Luis Estuardo Lombardi, Edgar Kestler, Obdulia Salic, Sergio Marroquin, Victor Argueta (Centro de Investigación Epidemiológica en Salud Sexual y Reproductiva-CIESAR, Hospital General San Juan de Dios); Walter Guerra (Instituto Nacional del Cáncer); Hesler Morales (Instituto Guatemalteco de Seguridad Social, Instituto Nacional del Cáncer); |
|  |
| 11.  Honduras: Annabelle Ferrera (Escuela de Microbiología, Universidad Nacional Autónoma de Honduras); |
|  |
| 12.  India: Asha Jain (Cancer Prevention and Relief Society Raipur); Sushil K Giri (Regional Cancer Center, Cuttack); Maheep Bhalla (JLN Hospital & Research Center,BSP, Bhilai); Bharat Patel (Lab One Raipur); PSA Sarma (BSP Hospital); Ravi Mehrotra, Mamta Singh (M.L.N Medical College, Allahabad); |
|  |
| 13.  Korea-South: Hai-Rim Shin, Jin-Kyung Oh (National Cancer Center); Shin Gwang Kang (Asan Medical Center); Dong-chul Kim (Kangnam St. Mary's Hospital); |
|  |
| 14.  Lebanon: Muhieddine Seoud (The American University of Beirut Medical Center); |
|  |
| 15.  Mexico: Isabel Alvarado-Cabrero (Instituto Mexicano del Seguro Social); Claudia Magaña-León, Rubén López-Revilla (Instituto Potosino de Investigación Científica y Tecnológica, AC); Cuauhtémoc Oros (Hospital Central Ignacio Morones Prieto, San Luis Potosí); |
|  |
| 16.  Mozambique: Carla Carrilho (Eduardo Mondlane University); |
|  |
| 17.  Nigeria: A.A.F. Banjo, F.B. Abdulkareem, A.O. Daramola, C.C. Anunobi, R.U. Anorlu (Lagos University Teaching Hospital Idi-Araba); Sani Malami, Ali Bala Umar (Faculty of Medicine, Bayero University); |
|  |
| 18.  Paraguay: Antonio Leopoldo Cubilla, Elena Kasamatsu, Francisco Perrota (Instituto de Investigaciones en Ciencias de la Salud, Universidad Nacional de Asunción); |
|  |
| 19.  Philippines: Efren j Domingo, Maria Julieta V. Germar, Jerico Thaddeus, P. Luna, Arnold M. Fernandez, Carolyn Zalameda Castro, Roslyn Balacuit (University of the Philippine College of Medicine General Hospital); |
|  |
| 20.  Poland: Andrzej Marcin Nowakowski (Medical University of Lublin); Robert Jach, Jolanta Orlowska-Heitzman, Monika Kabzinska-Turek, Paulina Przybylska, Marzena kula-Prykan (Jagiellonian University Medical College); |
|  |
| 21.  Portugal: Eugenia Cruz (Centro Regional de Oncologia Coimbra, Instituto Português de Oncologia); Ana Felix, Jorge Manuel Soares (Instituto Portugues de Oncologia de Lisboa Francisco Gentil); |
|  |
| 22.  Senegal: Cathy Ndiaye, Nafissatou Ndiaye Ba, Victorino Mendes (HOGGY stands for Hôpital Général de Grand Yoff ; DANTEC - Hôpital A. Le Dantec; FAC - Faculté de Médecine - Université Cheikh A. Diop); |
|  |
| 23.  Spain: Enrique Poblet (Hospital General Universitario de Albacete); August Vidal, Enric Condom (Hospital Universitari de Bellvitge); Lluís Eleuteri Pons Ferré, Patrícia Escrivà Beltri, Marylene Lejeune (Hospital de Tortosa Verge de La Cinta); Belén Lloveras, Emili Masferrer (Hospital del Mar); Julio Velasco Alonso, Cristina Pérez (Hospital San Agustin); Maria Alejo (Hospital General de L’Hospitalet); Laia Alemany, Francesc Xavier Bosch, Ignacio G. Bravo, Vanesa Camón, Gabriel Capellà, Xavier Castellsagué, Omar Clavero, Silvia de Sanjosé, Anna Esteban, José M. Godínez, Yolanda Florencia, Joellen Klaustermeier, Núbia Muñoz, Beatriz Quirós, Cristina Rajo, Sara Tous, Marleny Vergara (IDIBELL, Institut Català d’Oncologia-Catalan Institute of Oncology); |
|  |
| 24.  The Netherlands: Wim G.V. Quint, Anco C. Molijn, Daan T. Geraets, Núria Guimerà (DDL Diagnostic Laboratory); Chris J.L.M Meijer (Vrije Universiteit Medical Center); |
|  |
| 25.  UK: Ray Lonsdale (Norfolk & Norwich University Hospital NHS Foundation Trust); |
|  |
| 26.  USA: Wendy Cozen, Marc T. Goodman, Brenda Y. Hernández, Charles Lynch, Daniel B. Olson, Freda R. Selk (Cancer Center, Hawaii-Iowa); |
|  |
| 27.  Venezuela: Enrique López Loyo (Sociedad Venezolana de Patología); Victoria García Barriola, Mirian Naranjo de Gómez, Adayza Figueredo, Janira Navarro (Universidad Central de Venezuela). |
|  |
| 28.  The advisory committee members are: Chris J Meijer, Massimo Tommasino, Michael Pawlita, Wim Quint and Nubia Muñoz |
|  |
| 5.**HPV VVAP study group for anal site, updated June 2013:** |
|  |
| 1.      Australia: Gerard Vincent Wain, Catherine Jane Kennedy, Yoke-Eng Chiew (Gynaecological Oncology, Westmead Hospital); Raghwa Sharma (Department of Tissue Pathology and Diagnostic Oncology, University of Sydney and University of Western Sydney Westmead Hospital); |
|  |
| 2.      Bangladesh: Ashrafun Nessa, AJE Nahar Rahman, Mohammed Kamal (Bangabandhu Sheikh Mujib Medical University – BSMMU); Faruk Ahmed (Dhaka Medical College Hospital); |
|  |
| 3.      Bosnia Herzegovina: Ermina Iljazovic (University Clinical Center Tuzla BiH); |
|  |
| 4.      Chile: Rodrigo Prado, Carla Molina, rosa Muñoz (Centro de Oncología Preventiva, Facultad de Medicina, Universidad de Chile); Ximena Rodriguez, Marisol Guerrero,Virginia Leiva, Elsa Olave, Claudia Ramis, Viviana Toro (Hospital de San José); |
|  |
| 5.      Colombia: Raúl Murillo, Gustavo Adolfo Hernández Suárez, Carlos Eduardo Pinzón, Nubia Muñoz (Instituto Nacional de Cancerología); |
|  |
| 6.      Czech Republic: Václav Mandys (3rd Faculty of Medicine and University Hospital King’s Wineyards); Jan Laco (Faculty Hospital Hradec Kralove); |
|  |
| 7.      Ecuador: Leopoldo Tinoco (Hospital Oncológico Solca-Quito); |
|  |
| 8.      France: Christine Clavel, Philippe Birembaut, Veronique Dalstein (CHU de Reims, Laboratoire Pol Bouin/ INSERM UMR-S 903, REIMS); Christine Bergeron (Laboratoire Cerba, Department de Pathology, Cergy Pontoise); Massimo Tommasino (International Agency for Research on Cancer); |
|  |
| 9.      Germany: Karl Ulrich Petry, Alexander Luyten (Klinikum Wolfsburg); Michael Pawlita, Gordana Halec, Dana Holzinger (Department Genome Changes and Carcinogenesis, Heildelberg); |
|  |
| 10.  Guatemala: Luis Estuardo Lombardi, Edgar Kestler, Obdulia Salic, Sergio Marroquin, Victor Argueta (Centro de Investigación Epidemiológica en Salud Sexual y Reproductiva-CIESAR, Hospital General San Juan de Dios); Walter Guerra (Instituto Nacional del Cáncer); Hesler Morales (Instituto Guatemalteco de Seguridad Social, Instituto Nacional del Cáncer); |
|  |
| 11.  Honduras: Annabelle Ferrera (Universidad Autónoma de Honduras); Odessa Henríquez and Silvia Portillo (Instituto Nacional Cardiopulmonar en Tegucigalpa). |
|  |
| 12.  India: Asha Jain (Cancer Prevention and Relief Society Raipur); Sushil K Giri (Regional Cancer Center, Cuttack); Maheep Bhalla (JLN Hospital & Research Center,BSP, Bhilai); Bharat Patel (Lab One Raipur); PSA Sarma (BSP Hospital); Ravi Mehrotra, Mamta Singh (M.L.N Medical College, Allahabad); |
|  |
| 13.  Korea-South: Hai-Rim Shin, Jin-Kyung Oh (National Cancer Center); Shin Gwang Kang (Asan Medical Center); Dong-chul Kim (Kangnam St. Mary's Hospital); |
|  |
| 14.  Mali: Bakarou Kamate, Cathy Ndiaye (Hospital National DU Point G); |
|  |
| 15.  Mexico: Isabel Alvarado-Cabrero (Instituto Mexicano del Seguro Social); Rubén López-Revilla, Claudia Magaña-León (Instituto Potosino de Investigación Científica y Tecnológica, AC); Cuauhtémoc Oros (Hospital Central Ignacio Morones Prieto, San Luis Potosí); |
|  |
| 16.  Nigeria: Adekunbiola A.F. Banjo, F.B. Abdulkareem, A.O. Daramola, C.C. Anunobi, R.U. Anorlu (Lagos University Teaching Hospital Idi-Araba); Sani Malami, Ali Bala Umar (Faculty of Medicine, Bayero University); |
|  |
| 17.  Paraguay: Elena Kasamatsu, Antonio Leopoldo Cubilla, Francisco Perrota (Instituto de Investigaciones en Ciencias de la Salud, Universidad Nacional de Asunción, Instituto de Patología e Investigación); |
|  |
| 18.  Poland: Robert Jach, Jolanta Orlowska-Heitzman, Monika Kabzinska-Turek, Paulina Przybylska, Marzena kula-Prykan (Jagiellonian University Medical College); |
|  |
| 19.  Portugal: Ana Felix (Instituto Portugues de Oncologia de Lisboa Francisco Gentil); |
|  |
| 20.  Senegal: Cathy Ndiaye, Nafissatou Ndiaye Ba, Victorino Mendes (HOGGY stands for Hôpital Général de Grand Yoff ; DANTEC - Hôpital A. Le Dantec; FAC - Faculté de Médecine - Université Cheikh A. Diop); |
|  |
| 21.  Slovenia: Mario Poljak, Boris Pospihalj, Pavle Košorok (Institute of Microbiology and Immunology, Ljubljana); |
|  |
| 22.  Spain: Maria Alejo (Hospital General de l’Hospitalet); Mar Iglesias, Belén Lloveras, David Parés (Hospital del Mar); Laia Alemany, Francesc Xavier Bosch, Ignacio G. Bravo, Vanesa Camón, Xavier Castellsagué, Omar Clavero, Silvia de Sanjosé, Ion Espuña, Anna Esteban, José M. Godínez, Yolanda Florencia, Joellen Klaustermeier, Nubia Muñoz, Nati Patón, Beatriz Quirós, Cristina Rajo, Maëlle Saunier, Sara Tous, Marleny Vergara (IDIBELL, Institut Català d’Oncologia-Catalan Institute of Oncology, Barcelona); August Vidal, Enric Condom (Hospital Universitari de Bellvitge, Barcelona); Julio Velasco Alonso, Cristina Pérez (Hospital San Agustín); |
|  |
| 23.  The Netherlands: Wim G.V. Quint, Anco C. Molijn, Daan T. Geraets, Núria Guimerà (DDL Diagnostic Laboratory); Chris J.L.M Meijer (Vrije Universiteit Medical Center); |
|  |
| 24.  UK: Henry Kitchener, Godfrey Wilson (School of Medicine, University of Manchester); |
|  |
| 25.  USA: Wendy Cozen (Los Angeles, CA); Marc T. Goodman, Brenda Y. Hernández (Hawaii); Charles Lynch, Daniel B. Olson, Freda R. Selk (Iowa); Edyta C. Pirog (New York Hospital - Cornell Medical Centre). |
|  |
| 26.  The advisory committee members are: Chris J Meijer, Massimo Tommasino, Michael Pawlita, Wim Quint, and Nubia Muñoz |
