# Supplementary material for: HPV16 variants distribution in invasive cancers of the cervix, vulva, vagina, penis, and anus
Source: Cancer Med. 2016 Sep 21;5(10):2909–19. doi: 10.1002/cam4.870 (PMC5083745; doi:10.1002/cam4.870)
Supplement: Supplementary file 2 — Figure S2. Mid‐point rooted HPV16 best‐known maximum likelihood phylogenetic tree, constructed using 109 unique full‐length genome sequences. HPV16 lineages are classified into four variants: A, B, C, and D. Bootstrap values above 700 are displayed closed to the corresponding node. GenBank accession numbers are given for all entries. [file CAM4-5-2909-s002.pdf]

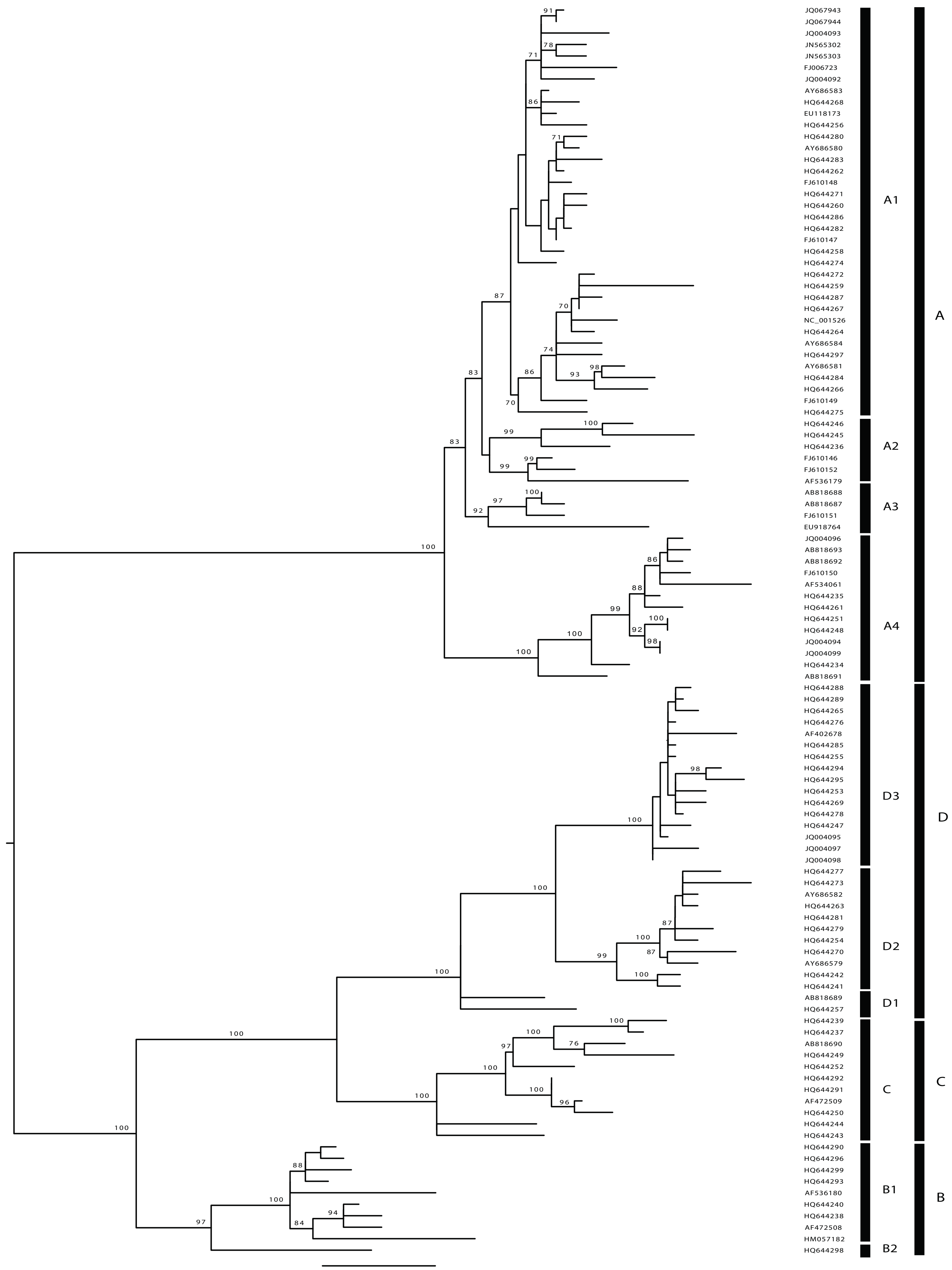

Figure S2: Mid-point rooted HPV16 Best-known maximum likelihood phylogenetic tree, constructed using 109 unique full length genome sequences. HPV16 lineages are classified into four variants: A, B, C and D. Boostrap values above 70 are displayed closed to the corresponding node. GenBank accession numbers are given for all entries.
